# A *de novo* substructure generation algorithm for identifying the privileged chemical fragments of liver X receptorβ agonists

**DOI:** 10.1038/s41598-017-08848-4

**Published:** 2017-09-11

**Authors:** He Peng, Zhihong Liu, Xin Yan, Jian Ren, Jun Xu

**Affiliations:** Research Center for Drug Discovery, School of Pharmaceutical Sciences and School of Life Sciences, Sun Yat-Sen University, 132 East Circle at University City, Guangzhou, 510006 China

## Abstract

Liver X receptorβ (LXRβ) is a promising therapeutic target for lipid disorders, atherosclerosis, chronic inflammation, autoimmunity, cancer and neurodegenerative diseases. Druggable LXRβ agonists have been explored over the past decades. However, the pocket of LXRβ ligand-binding domain (LBD) is too large to predict LXRβ agonists with novel scaffolds based on either receptor or agonist structures. In this paper, we report a *de novo* algorithm which drives privileged LXRβ agonist fragments by starting with individual chemical bonds (*de novo*) from every molecule in a LXRβ agonist library, growing the bonds into substructures based on the agonist structures with isomorphic and homomorphic restrictions, and electing the privileged fragments from the substructures with a popularity threshold and background chemical and biological knowledge. Using these privileged fragments as queries, we were able to figure out the rules to reconstruct LXRβ agonist molecules from the fragments. The privileged fragments were validated by building regularized logistic regression (RLR) and supporting vector machine (SVM) models as descriptors to predict a LXRβ agonist activities.

## Introduction

Liver X receptorβ (LXRβ, also known as NR1H2) is a nuclear receptor, which is considered as the core of modern pharmacology, and the promising therapeutic target for lipid disorders, atherosclerosis, chronic inflammation, autoimmunity, cancer and neurodegenerative diseases^1, 2^. But, LXRβ ligand-binding domains (LBDs) have a big binding pocket, which tolerates diverse sizes and shapes of ligands. This makes difficult to predict LXRβ ligand structures with novel scaffolds based upon known receptor or ligand structures^3^. Thousands of natural or synthetic LXR agonists have been reported. Conventional approaches were also tried to predict LXR agonists^4–7^. In current studies, we were motivated to figure out privileged LXRβ agonist fragments from the known LXRβ agonists to guide fragment-based^8^ LXRβ agonist design and discovery.

There are many ways to define or derive structural fragments (substructures) from a chemical structure library, such as, maximal common substructure (MCSS) algorithm^9^, fingerprint algorithms^10^, scaffold-based classification approach (SCA)^11^, atom center fragments^12, 13^, etc. These approaches were based upon empirically or algorithmically pre-defined rules^14, 15^ and, the resulting substructures could be subjective. To build predictive SAR models, we need substructures that are statistically representative in a chemical structure library and related to the concerned activity.

Over the last decade, subgraph mining algorithms were developed and applied in QSAR modeling. Dehaspe and colleagues^16^ used a subgraph discovery algorithm to predict the toxicity of a compound based upon its chemical structure. Yan and Han developed the gSpan program for subgraph mining^17^. Huan and colleagues addressed the isomorphism problem in the subgraph mining process^18^. Kuramochi and coworkers also developed a subgraph discovery program^19^. Borgelt and colleagues developed MoSS for subgraph mining^20, 21^. Meinl and co-workers developed the ParMol package for subgraph mining^22^. Wang and colleagues paralleled a subgraph mining algorithm with the CUDA technology^23^. Most recently, Khashan and co-workers used the subgraph mining approach in QSAR Modeling to predict compound toxicity^24^. Shao and colleagues used a subgraph mining technology to identify common functional groups to predict drug adverse effects^25^.

These algorithms were tested on smaller data sets ranging from 10 K to 100 K compounds, some of them were tested on artificially generated data^17, 19, 26, 27^. Nowadays, chemical structure data (such as ZINC, one of the largest databases for medicinal chemistry, contains approximately 21 million compounds) grow rapidly^28^. Our studies revealed that a conventional subgraph (substructure) mining algorithm would encounter a huge computational challenge when it was tested on a million-compounds database due demanding huge memory for the isomorphism checking (more than 128 GB). Those subgraph mining algorithms elected substructures based upon a minimal support threshold, which was determined by trial-and-errors. Raising the threshold would be at the risk of losing substructures, which were related to the activity. Lowering the threshold would be at the risk of introducing too many trivial substructures, which reduced the prediction accuracy for lowering signal-to-noise ratio. Consequently, the classification accuracies were around 70%^24^. Moreover, most of the previous subgraph mining approaches did not interpret the subgraph chemistry, which should be of interest to chemists. Khashan and colleagues did study the relationship of their substructures and toxiphores. But, these fragments were derived without considering the chemical integrity (such as, an aromatic ring was broken in the middle of the ring).

**Figure 1:**
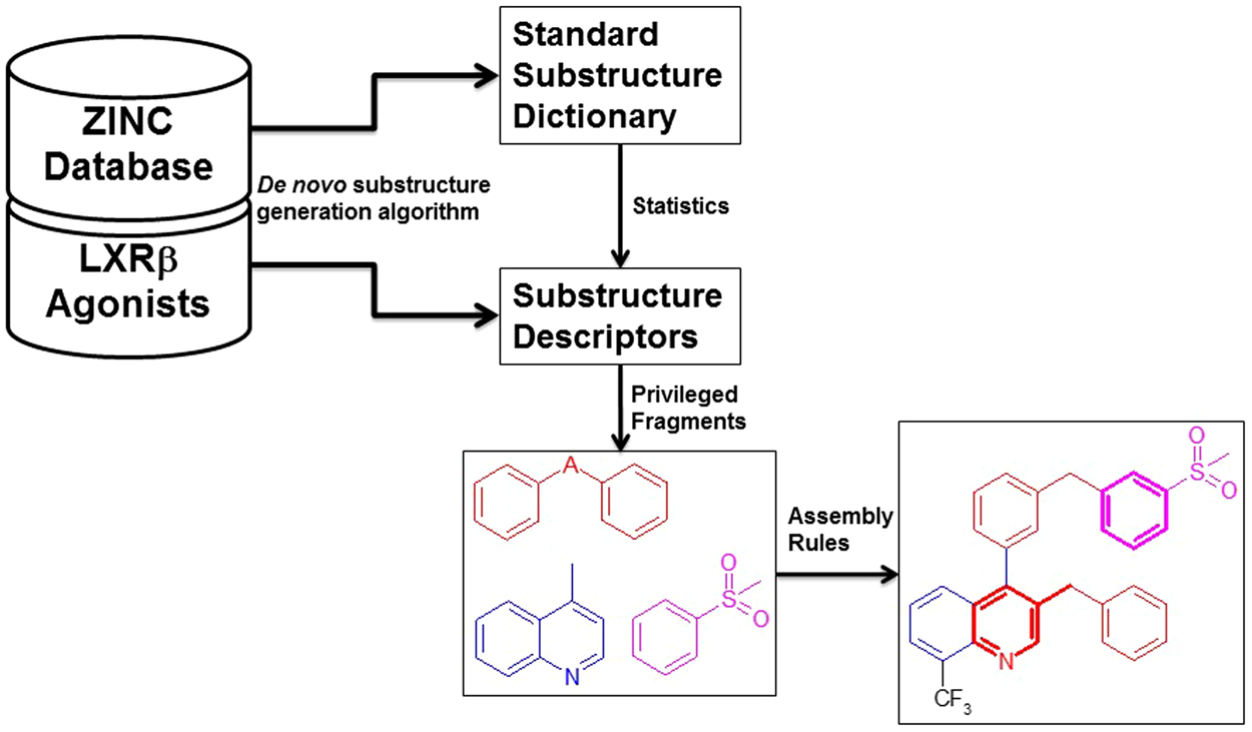
The flow-chart for using *de novo* substructure generation algorithm to discover LXRβ agonist privileged fragments and elucidate the assembly rules.

In order to solve these problems, we propose a new *de novo* substructure generation algorithm (DSGA), which discovers substructures from a chemical structure library with improved substructure mining strategies:

1. To avoid generating too many trivial substructures and reducing the memory requirements for the isomorphism checking, we coded growing subgraphs with linear notations (subIDs, see Fig. 1). The advantage of the subID linear notation is that the isomorphism checking can be done by a substring search instead of a subgraph search, which demands memory and computing resource.
2. When substructures were generated with a depth-first search strategy, the computing complexity could grow exponentially. Therefore, we developed a strategy to prune the depth-first search tree to converge the results. The algorithm only grows the nodes with the maximal substructure on the search tree, other branches in the tree will be pruned. To examine if a substructure is a maximal substructure, the GMA algorithm^9^ was employed to exclude isomorphic or homomorphic substructures.
3. The further integrity checking was applied to ensure the chemical relevant of the maximal substructures.

Conventionally, the library under investigation is used to find substructures as a structural descriptor vector for a molecule in a compound library. However, electing substructures only based on the minimal support threshold may include too many trivial substructures or missed the under-supported substructures that are still strongly related to the activity. This problem can be resolved by taking chemical and biological background knowledge (chemical functional groups or synthetic feasibility, and biological activities) into account. We ran the *de novo* algorithm on the ZINC database (the commonly recognized database in medicinal chemistry) to gain maximal substructures as the background knowledge to produce substructures as privileged fragments for LXRβ ligands. The knowledge is in the form of a standard substructure dictionary (SSD). With the SSD, a substructure can still be elected as a privileged fragment even if its population is below the “minimal support threshold”; a substructure can still be excluded even if its population is much higher than the “minimal support threshold”. The gold criterion is the relation between the substructure and the concerned property.

With DSGA, a compound library can be converted into a set of substructure descriptor vectors or an *m* x *n* matrix (*m* is the number of maximal frequent substructures, and *n* is the number of compounds in the library). If the matrix is associated with activities, a regularized logistic regression (RLR) model^29^ or other machine learning models can be constructed to predict the activity for a new compound based on its chemical structure.

Comparing with previous studies, we emphasize more on gaining new chemical insights from the substructure mining algorithm. In fragment-based drug discovery (FBDD)^30^, key questions to be answered are what are the fragments for a drug lead, and what are the rules to combine these fragments. In this work, we present an example on how one can answer these questions by applying a *de novo* substructure generation algorithm (Fig. 1). This work can be applied for analyzing privileged fragments for the ligands against other biological targets.

## Results and Discussion

### *De novo* substructure generation process with a pruning strategy

The pruning strategy significantly reduced the number of substructures discovered from the three testing libraries(﻿LXRβ, PPARα, and VR libraries). The ratios of total-substructures/pruned-substructures are 113, 105, and 114 for the LXRβ library (634 compounds), PPARα library (606 compounds), and VR library (619 compounds), respectively. This means the pruning strategy improves the performance more than one hundred times. The pruning strategy is particularly important when *de novo* substructure generation algorithm (DSGA) is used in a big compound library (such as a library with more than 100 K compounds). In our studies, the LXRβ library has only 161 frequent substructures, a program without pruning strategy has to check 18,170 substructures; for the VR library (83 frequent substructures) checking 9,439 substructures; and for the PPARα library (114 frequent substructures) checking 12,021 substructures. This costs not only computing time, but exhausts so much memory that an algorithm cannot continue the calculation due to no enough memory.

With the pruning strategy, we, for the first time, are able to generate substructures from the ZINC database^31^, which has approximately 9.1 million drug-like compounds. The algorithm discovered 51,770 substructures from the ZINC database. The number of substructures increased exponentially before the first 10 K structures of the ZINC database were scanned, and the number significantly slowed down because most of the maximal substructures had been discovered. This suggested that the structural diversity of substructures is limited in the currently explored chemical space (Figure S1).

**Figure 2:**
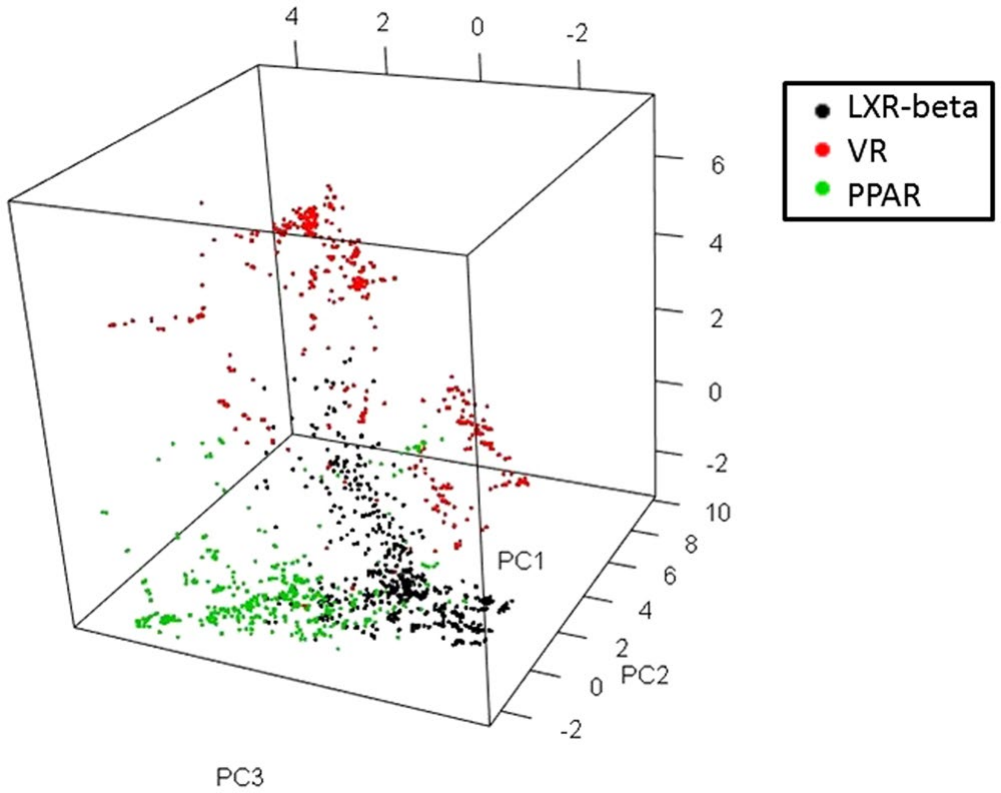
LXRβ, PPARα and VR libraries were discriminated by the frequent substructure descriptors derived from the ZINC library.

By using the frequent substructuresthat were generated from the ZINC library as descriptors, we were able to discriminate three focused compound libraries associated with three different biological targets (LXRβ, PPARα, and VR) with principal component analyses (PCA) as depicted in Fig. 2.

### Substructures used for predicting activities with the RLR approach

**Figure 3:**
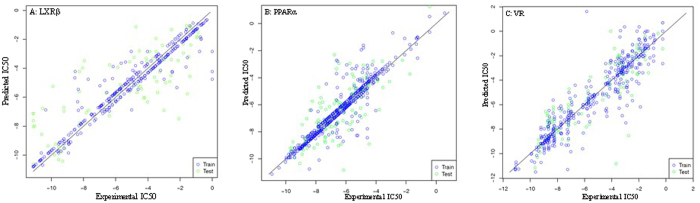
The performances of three substructure-based SVM regression models. (**A**) LXRβ, (**B**) PPARα, (**C**) VR.

One way to examine the quality of DSGA is to study the relations between the substructures and bioactivities. 51,170 substructures were derived from the ZINC database (9,107,119 compounds), and used as the SSD for building RLR classification models. Again, three compound libraries for LXRβ, PPARα, and VR, were studied for SSD-based RLR classification modeling to predict the activities against LXRβ, PPARα, and VR. Figure 3 demonstrates the prediction capacities of the SVM models.

The RLR classification model performances are summarized in Table 1. The ratio of splits between train and validation data is 2:1.

**Table 1:** The RLR classification model performances.

Targets	Sensitivity	Sensitivity	ROC	Accuracy
LXRβ	0.927	0.759	0.930	0.836
PPARα	0.872	0.765	0.883	0.839
VR	0.932	0.706	0.916	0.868

These results demonstrated that the substructures discovered by DSGA are objective structural descriptors for RLR classifications.

### Substructures used for predicting activities with SVM regression

Regression modeling requires reducing the number of descriptors in order to avoid high computational costs. A subset of the SSD was derived based upon the population tuning points for a specific compound library. In Fig. 5, the X-axis stands for the substructure, the Y-axis stands for the frequency of the corresponding substructure in a compound library. This plot demonstrates the distributions of the substructures in the VR, LXRβ, and PPARα libraries. The curves begin to flatten at the frequency of 40, where the LXRβ and PPARα libraries can adopt 3,000 substructures, and the VR library can adopt 4,375 substructures as their structural descriptors.

The SVM regression models were built for the LXRβ, PPARα, and VR libraries with 277, 484, and 495 training compounds. The predictive models were validated with 5-fold validation processes. The performances were measured using the average mean square errors (MSE) and Pearson Correlations as listed in Table 2.

**Table 2:** Result of pIC50 prediction.

Target	MSE	Correlation
LXRβ	3.51	0.78
PPARα	2.07	0.73
VR	1.76	0.89

These results conclude that the substructures are highly related to the bioactivities. The prediction accuracies of regression models were not very high due to the paradox of predictivity versus diversity (that is, the greater the chemical diversity of the investigated compounds, the smaller the chance that SAR models exist and can be uncovered)^15^. The limit of this approach is that it is difficult for a common structure fragment descriptor to distinguish tiny structural differences among molecules. However, the advantage of this approach is that privileged structural fragments can be derived from these models.

### Identifying privileged fragments for privileged scaffold exploration

The substructures used in SVM models were scored with a privileged fragment index (PFI) as the following,

1 $${\rm{PFI}}({i})={f}_{i}\frac{{a}_{i}}{T}$$

where, *f*_i_ is the population of the *i*th substructure appearing in a given compound library, *T* is the total number of compounds in the library, and *a*_*i*_ is the number of active compounds in the library. All the substructures used in the SVM models for the LXRβ, PPARα, and VR libraries were sorted in descending order of the PFIs (Supplementary Materials).Privileged fragments for the LXRβ ligands found by DSGA are listed in Table 3.

**Table 3:** Privileged fragments for the LXRβ ligands.

No.	Privileged fragment	Active compounds	EC_50_ (μM)	Most active compound
A		108	0.011~4.17	
B		110	0.002~3.3	
C		74	0.023~5.4	
D		68	0.011~3.4	
E		33	0.004~1.57	
F		24	0.049~3.3	
G		11	0.001~1.5	
H		21	0.076~3.16	
I		66	0.006~8.0	
J		57	0.004~9.7	

### Rules to construct LXRβ agonists from the privileged fragment

#### Rule 1

**Figure 4:**
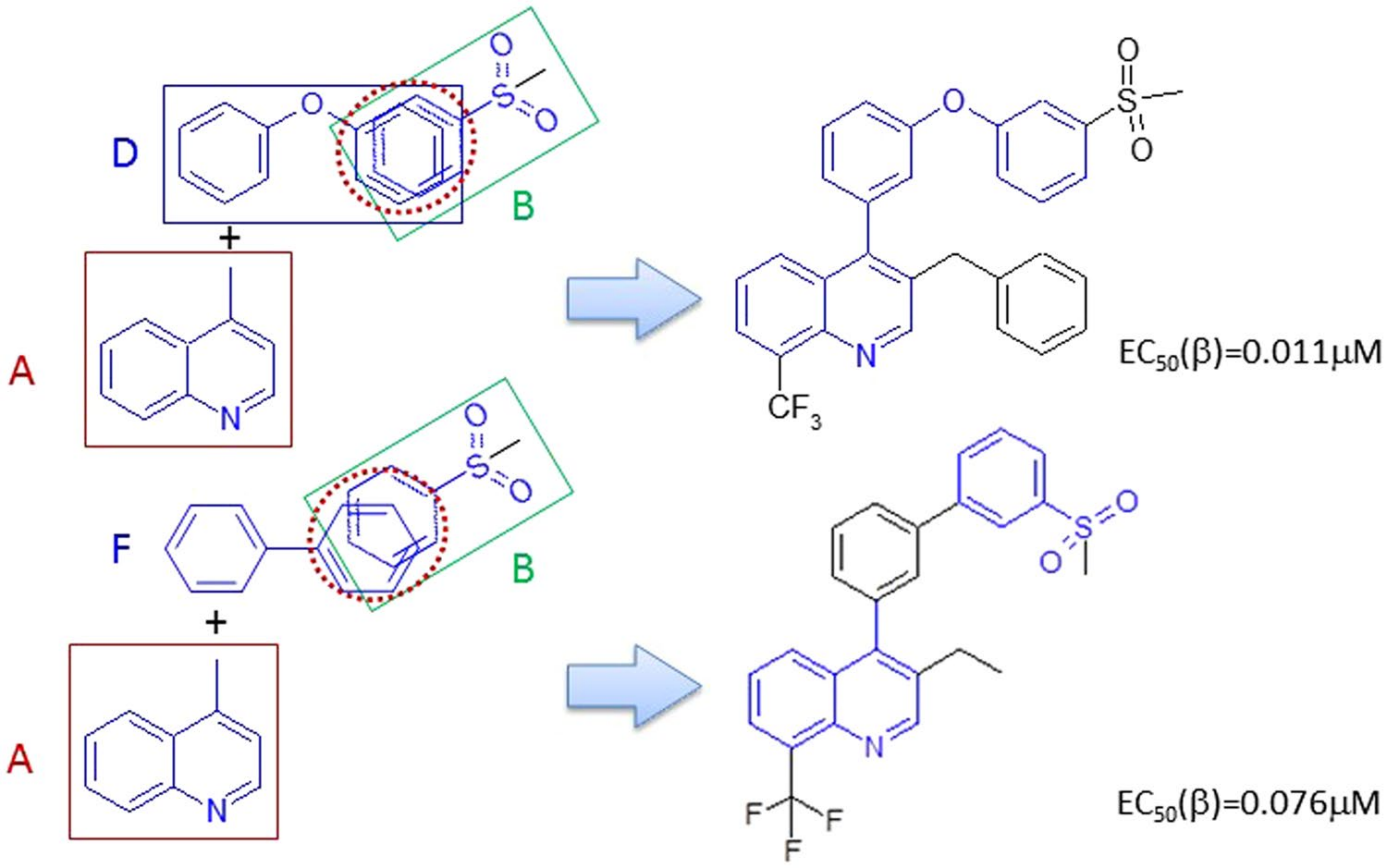
The schema of Rule 1.

Fragments D and F are linkers connecting A and B. There were 36 LXRβ ligands made through this rule. There were 23 such ligands linked through the MF substructure D, other 13 ligands were linked through the MF substructure F (details can be found in the supplementary material SM Table 1). The linker MF substructure D can produce more active ligands. The schema of Rule 1 is depicted in Fig. 4.

#### Rule 2

**Figure 5:**
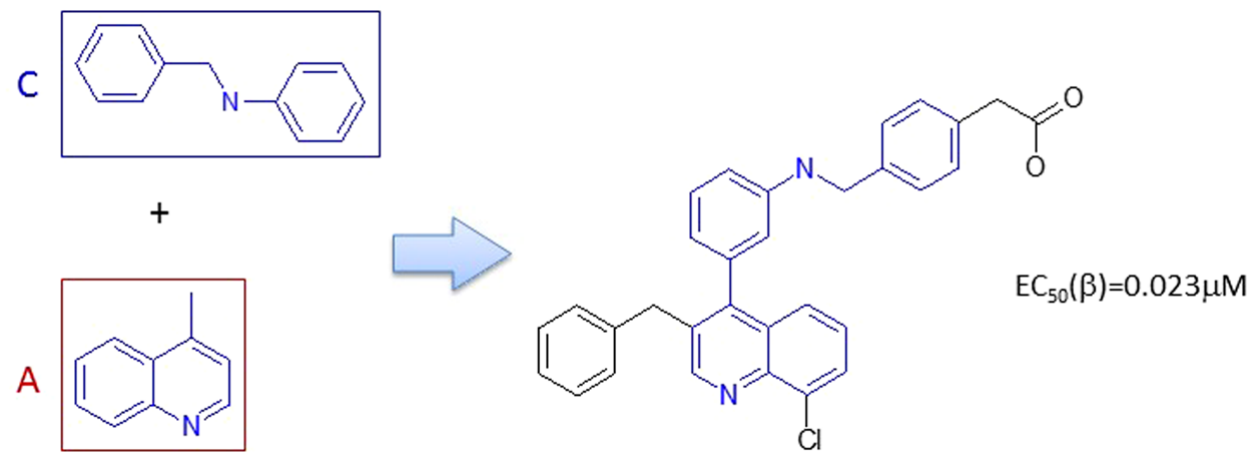
The schema of Rule 2.

The MF substructures A and C connect through direct covalent binding to make LXRβ ligands. The Fragment C is modified to allow any heavy atom at the position of the nitrogen atom. Thus, we got 31 LXRβ agonists based upon this rule. Fragment C has two classes of bioisosteres (the hetero atom linker can be nitrogen or oxygen), which do not significantly change the binding affinity. It seams that Fragment A cannot be simplified, and it is critical to maintain an acidic polar group at the terminal of Fragment C (details can be found in supplementary material SM Table 2). The schema of Rule 2 is demonstrated in Fig. 5.

#### Rule 3

**Figure 6:**
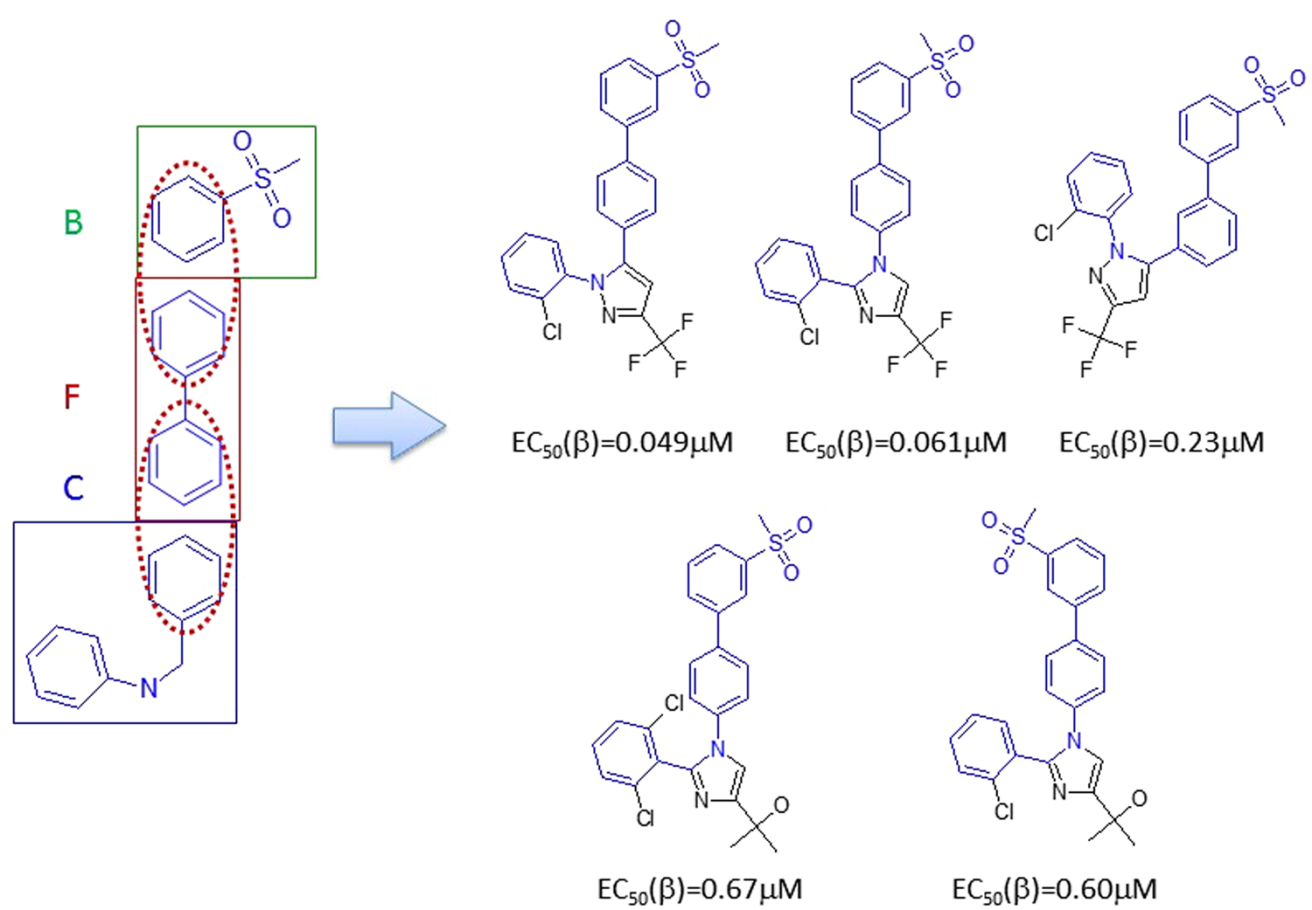
LXRβ ligands created by merging Fragments B, C and F (Rule 3).

Fragments B and C can be linked to form an LXRβ agonist. This combination can also be viewed as Fragment F merges with Fragments B and C. Although, only 5 LXRβ agonists were discovered, there are many opportunities to explore (Fig. 6).

#### Rule 4

**Figure 7:**
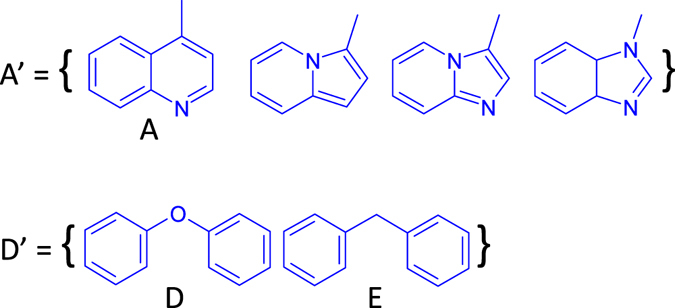
Bioisostere definitions for Fragments A’ and D’.

**Figure 8:**
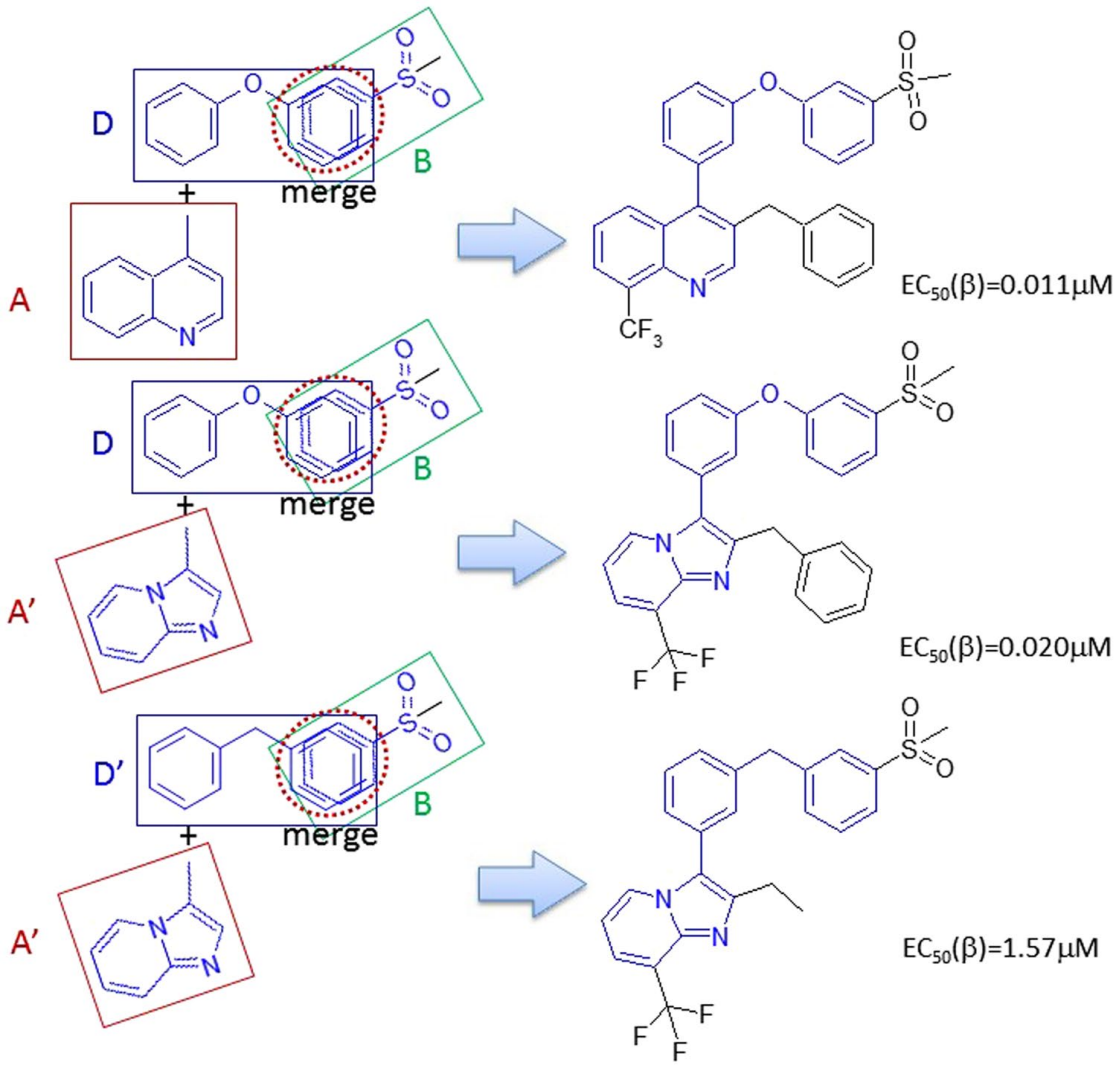
LXRβ ligands generated by Rule 4.

**Figure 9:**
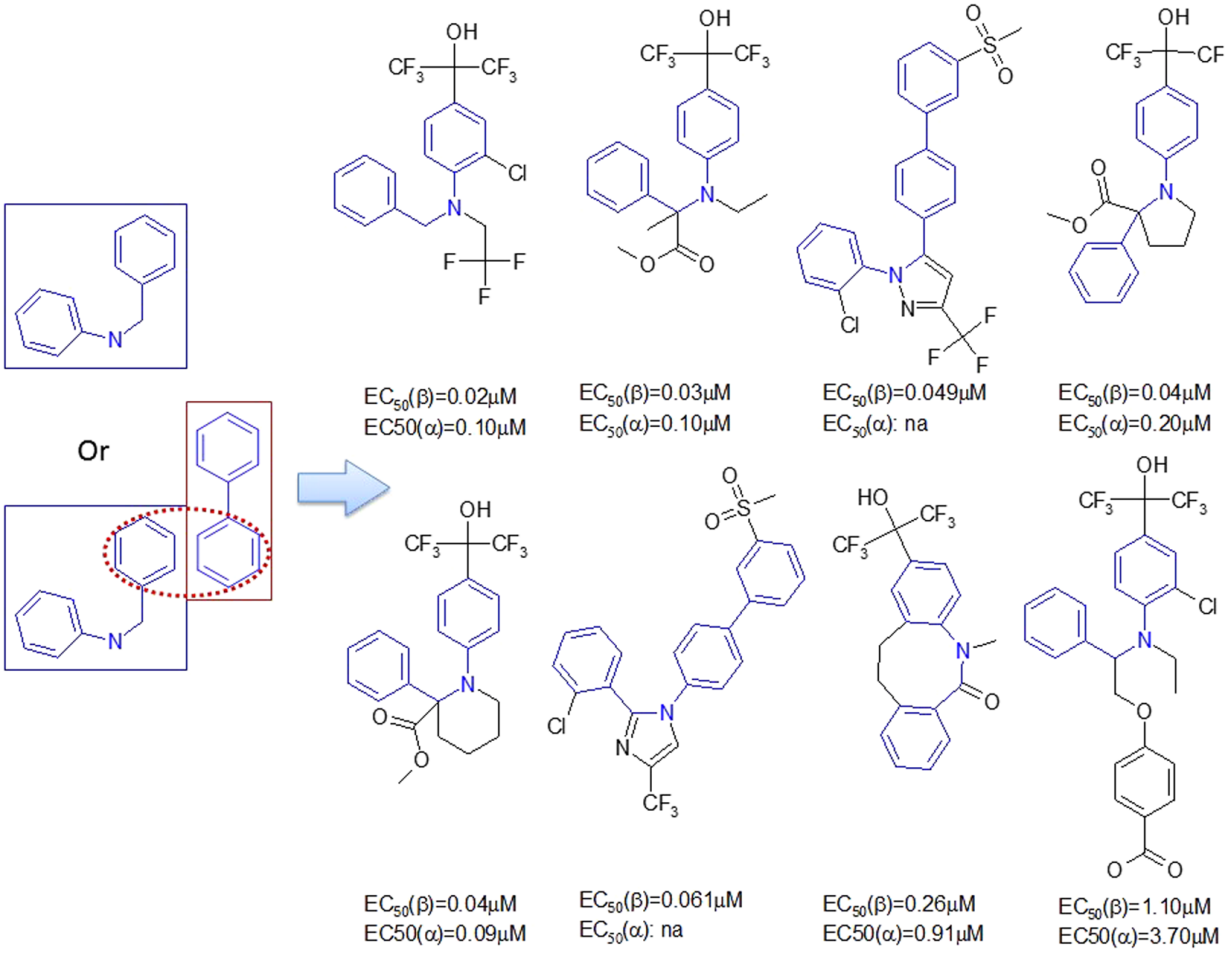
LXRβ agonists constructed by Rule 5.

Typical LXRβ agonist constructing cases are demonstrated in Figs 7 and 8. By inspecting the data set, we recognize that A and D have bioisosteres. Therefore, we define Fragments A′ and D′ as shown in Fig. 9. LXRβ agonists can be created by merging Fragments A’, B, and D’. Fragment A’ connects to Fragment D’, and Fragment D’ merges with B at the aromatic rings. This results in 66 LXRβ ligands with EC50 values ranging between 0.011 and 3.40 μM (details can be found in supplementary material SM Table 3 (Rule 4)). In this case, Fragment D’ is a linker to connect Fragments A’ and B.

#### Rule 5

Fragment C itself can be an LXRβ agonist scaffold. It can also be merged with Fragment F. The typical agonists are listed in supplementary material (details can be found in supplementary material SM Table 4 (Rule 5)). Typical ligands and their activities are depicted in Fig. 9.

#### Rule 6

**Figure 10:**
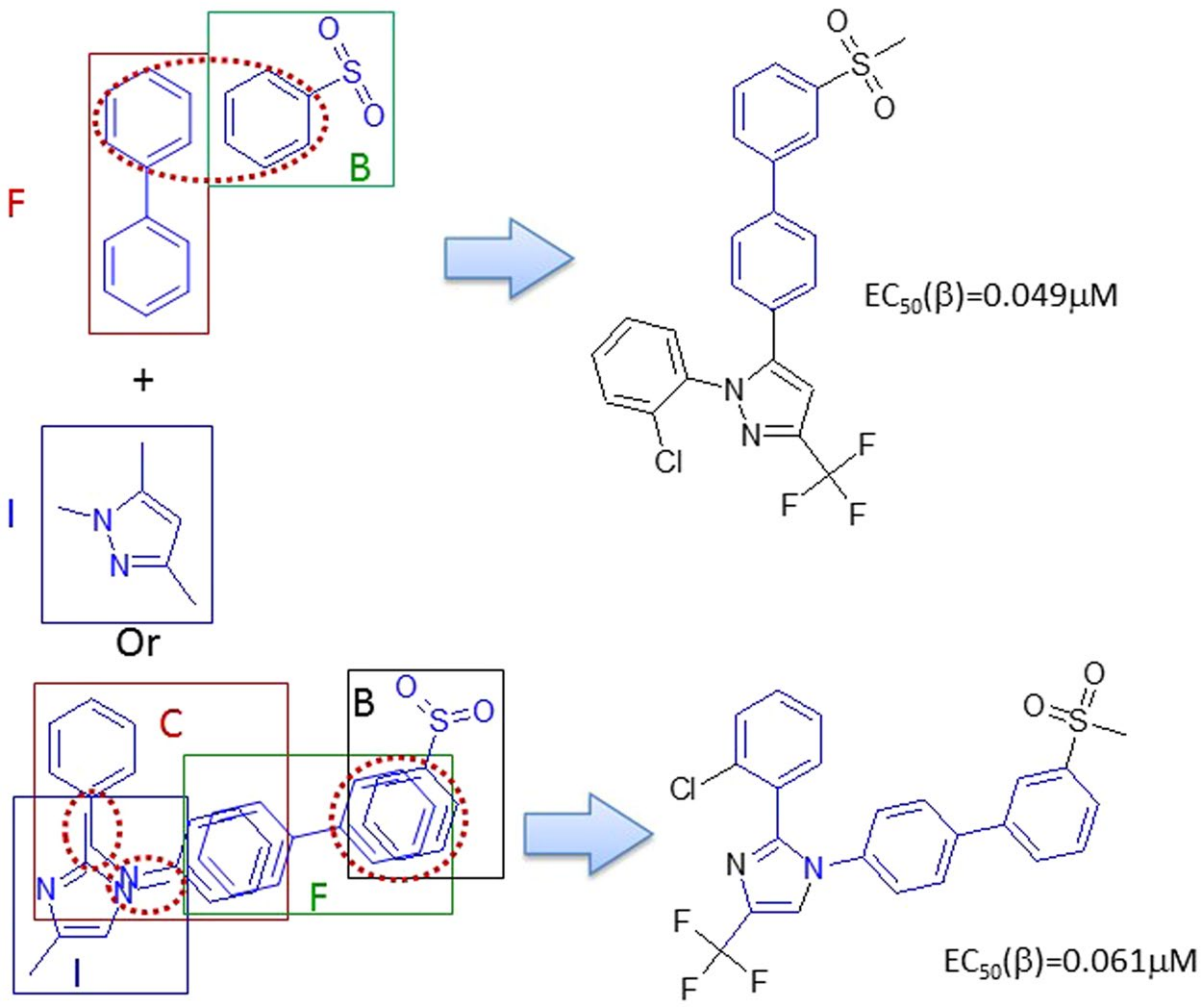
Scaffold constructed by rule 6.

Fragment I itself can form a star-shaped scaffold with a pentagon for an LXRβ agonist. It may merge with Fragments F and B, or C. 66 LXRβ agonists were constructed with this rule as shown in Fig. 10 (SM Table 5: Rule 6).

#### Rule 7

**Figure 11:**
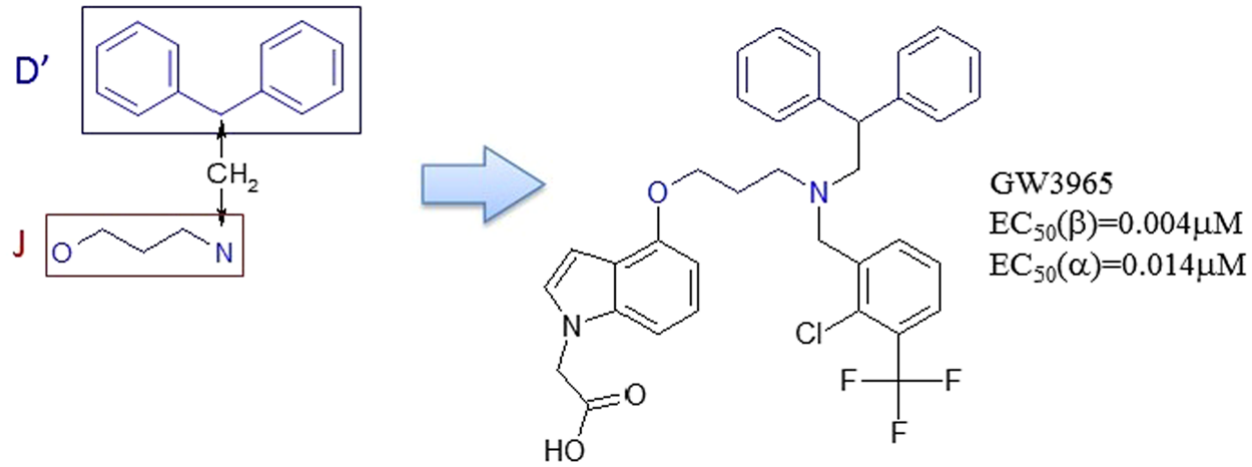
Scaffold constructed by Rule 7. The structure on the right is GW3965.

Fragments J and D’ can form a new scaffold by a methylene linker as shown in Fig. 11. These ligands are listed in supplementary material (SM Table 6: Rule 7).

#### Rule 8

**Figure 12:**
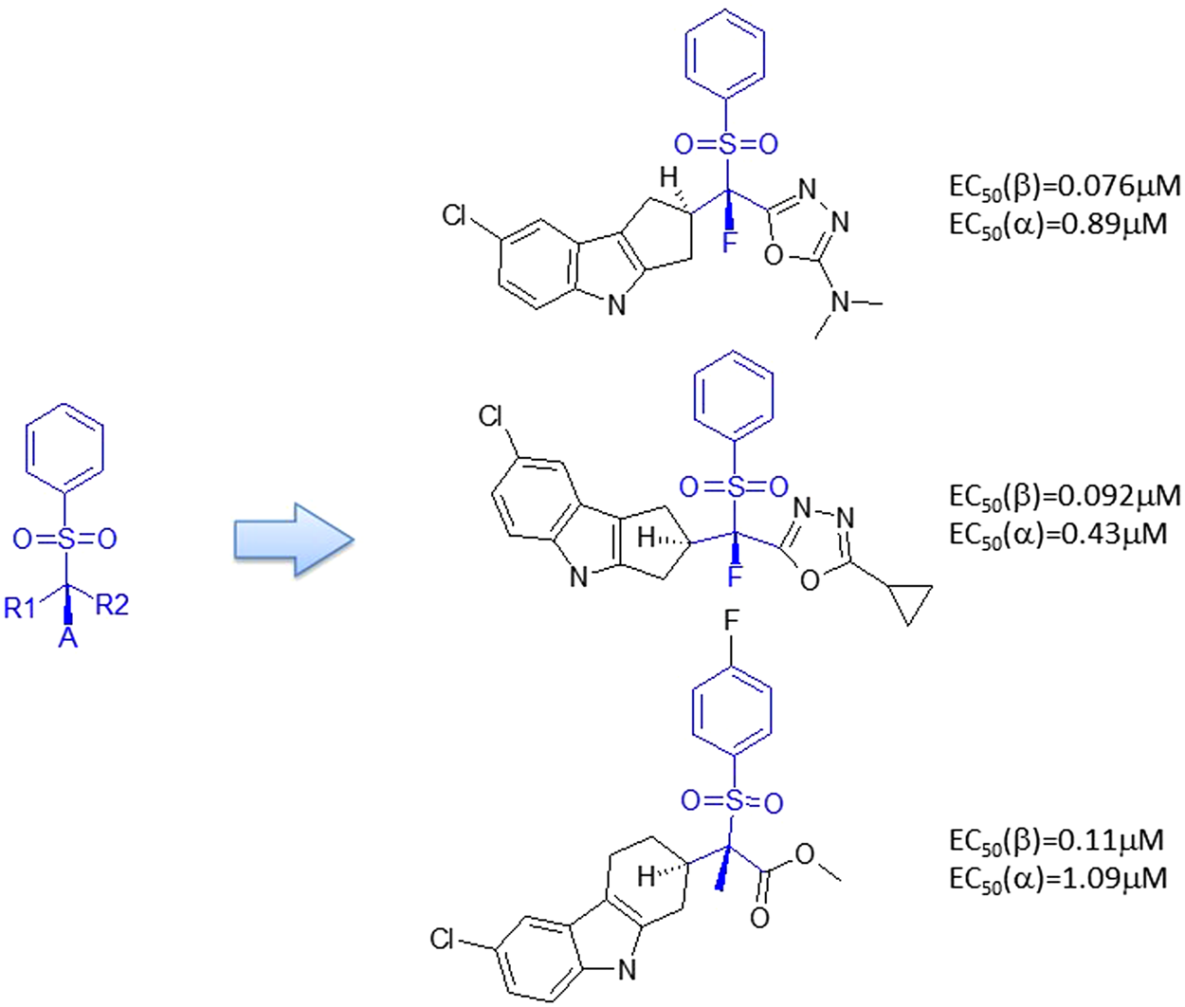
Scaffold constructed by Rule 8.

Fragment H forms a scaffold without connected with any other fragments reported in Table 3. These ligands are listed in supplementary material (SM Table 7: Rule 8). Typical examples are depicted in Fig. 12.

**Figure 13:**
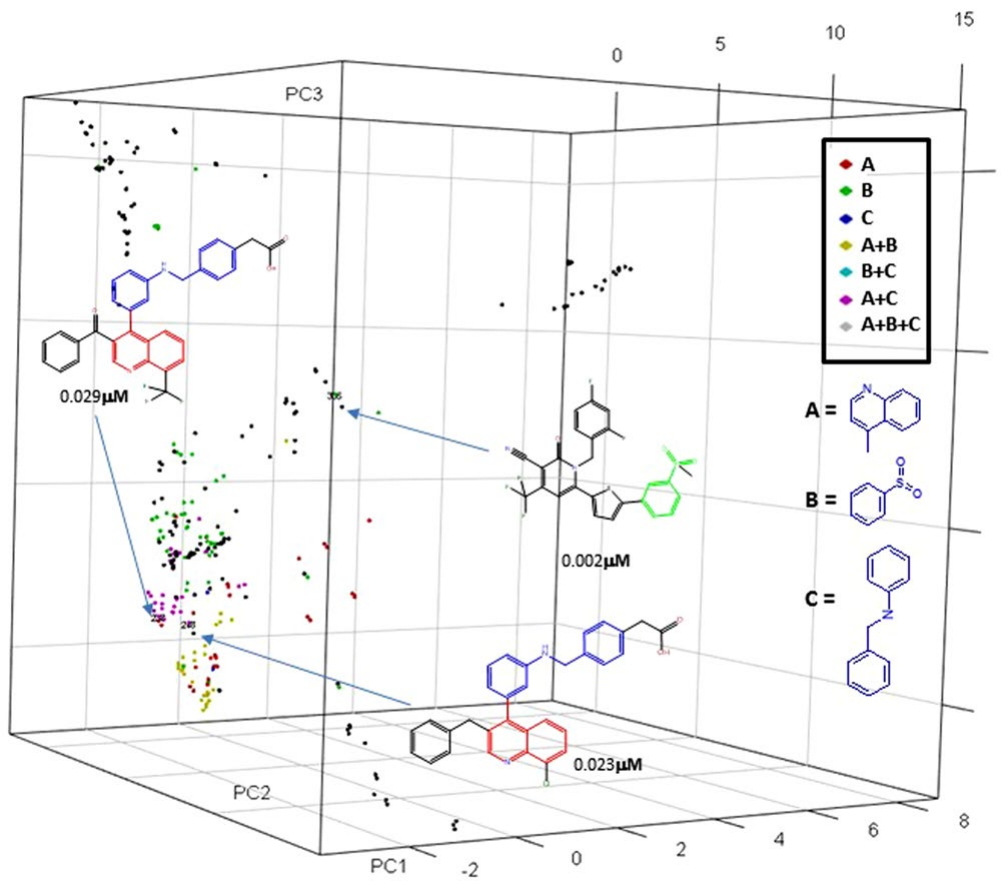
PCA plot for the compounds in the LXRβ library using the frequent fragment descriptors derived from ZINC database. Privileged fragments and their combinations are coded in different colors. The compounds with the same color are aggregated.

With the frequent fragment descriptors derived from ZINC database, the compounds in LXRβ library are depicted in three-dimensional space by means of PCA as shown in Fig. 13.

Figure 13 demonstrates that the privileged fragments (Table 3) and their combinations are capable at discriminating compounds with similar scaffolds.

### Experimental results

**Figure 14:**
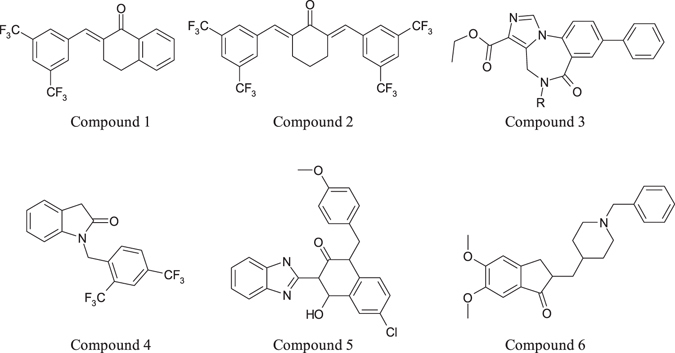
The experimentally confirmed LXRβ agonistic compounds found based upon the rules of privileged fragments and their combinations. At compound 3, R is a halogenated long-hydrocarbon substituent.

Based upon the above-mentioned rules, we selected compounds from our in-house compounds library for biological assays. Six compounds are found active against LXRβ in cell-based LXRβ agonistic assays. The compounds are listed in Fig. 14.

**Figure 15:**
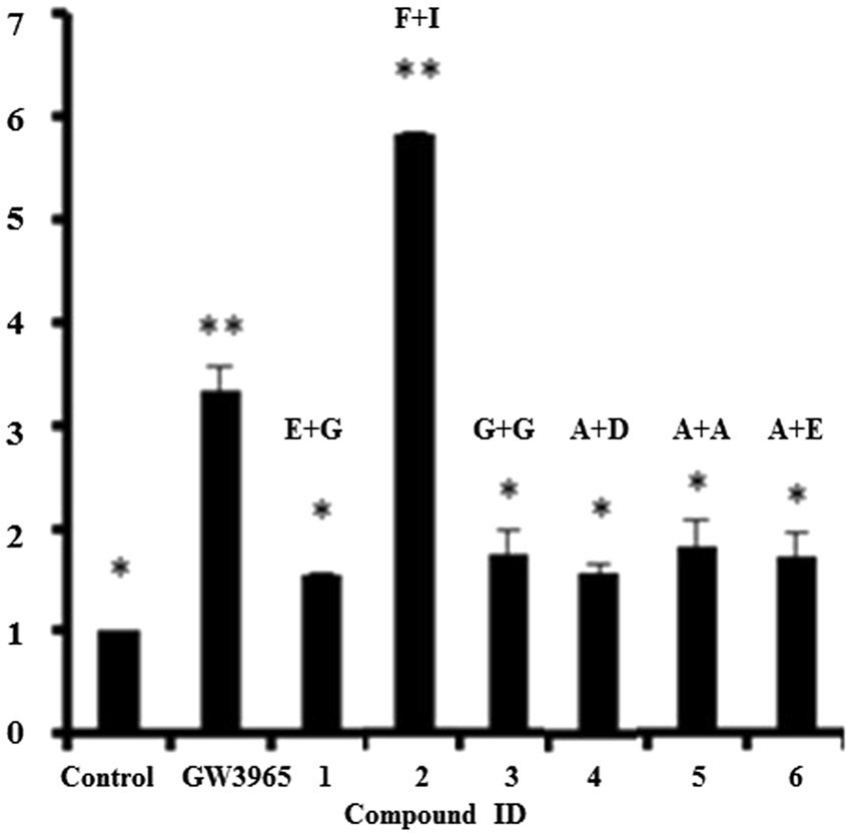
The activities of the confirmed LXRβ agonistic compounds and their fragment combination patterns. The letters above the bars represent the privileged fragments discovered by our algorithm. The structure of GW3965 is depicted in Fig. 11.

The activities of the confirmed LXRβ agonistic compounds are depicted in Fig. 15. GW3965 is for positive control. The compound 2 activated LXRβ significantly, the EC50 of which is 2.66 µM.

### Discussion

Over the past decades, many substructure generation approaches have been reported, such as empirical search keys^32^, algorithm-based atom center fragments^13, 33, 34^, fingerprints (http://www.daylight.com/)^15, 35^. *De novo* substructures are derived by algorithms with a given minimal support threshold (popularity threshold). It is difficult to determine the threshold. The lower threshold results in too many trivial substructures, and the higher threshold results in potentially losing substructures that have strong relations with the activity. Another concern is that these substructure mining algorithms produce partial substructures (incomplete rings or aromaticity). In essence, these frequent substructures need to be refined with chemical and biological knowledge. Our approach is developed to resolve these problems. The features of our algorithm are summarized as follows:

1. We introduce a linear notation to encode growing substructures into strings which are used to filter out most of isomorphic substructures. This technique converted the atom-by-atom isomorphism checking process to a string comparison, dramatically reducing the computing complexity, and allowed us to run the frequent substructure discovery algorithm on “big” data (over ten million compounds level).
2. Thus, we have derived the standard MF substructure dictionary (SMFSD) for selecting substructures for a small compound library to keep relevant chemical and biological substructures and exclude trivial substructures. We proved that this method improved the accuracies of the predictions (Table 1).
3. Most of the previous substructure mining algorithms did not interpret the chemistry of the frequent substructures. Based on our method, we can derive privileged structures for a focused compound library, and figure out the rules to assembly these substructures (or building blocks for a drug lead). These rules can be used to guide a medicinal chemist in synthetic design for a drug target.
4. Regarding the comparison of our approach with the matched molecular pairs (MMP) approach^36^, MMP method focuses on identifying every pair of molecules that differ only by a particular, well-defined, structural transformation. Our method, however, focuses on gaining new chemical insights from the substructure mining algorithm without predefined chemical substructures.

## Methods and Materials

### Molecular graph

A compound is represented in a molecular graph (MG). MG is an object consisting of an atom list, a bond-list, and a molecular attribute list. Each atom in the atom list is an object containing atomic attributes, such as, atom ID, atomic number, mass, charge status, binding adjacency etc. Each bond in the bond list is an object containing chemical bond attributes, such as, bond ID, bond types, two binding atom IDs, and stereo description, etc. The molecular attribute list holds data including molecular ID, weight, name, activities, and other properties. The MG external representation is MOL format. A compound library consists of a number of small molecules represented in MGs. In graph theory, a compound library is a molecular graph database.

### Maximal substructure tree

**Figure 16:**
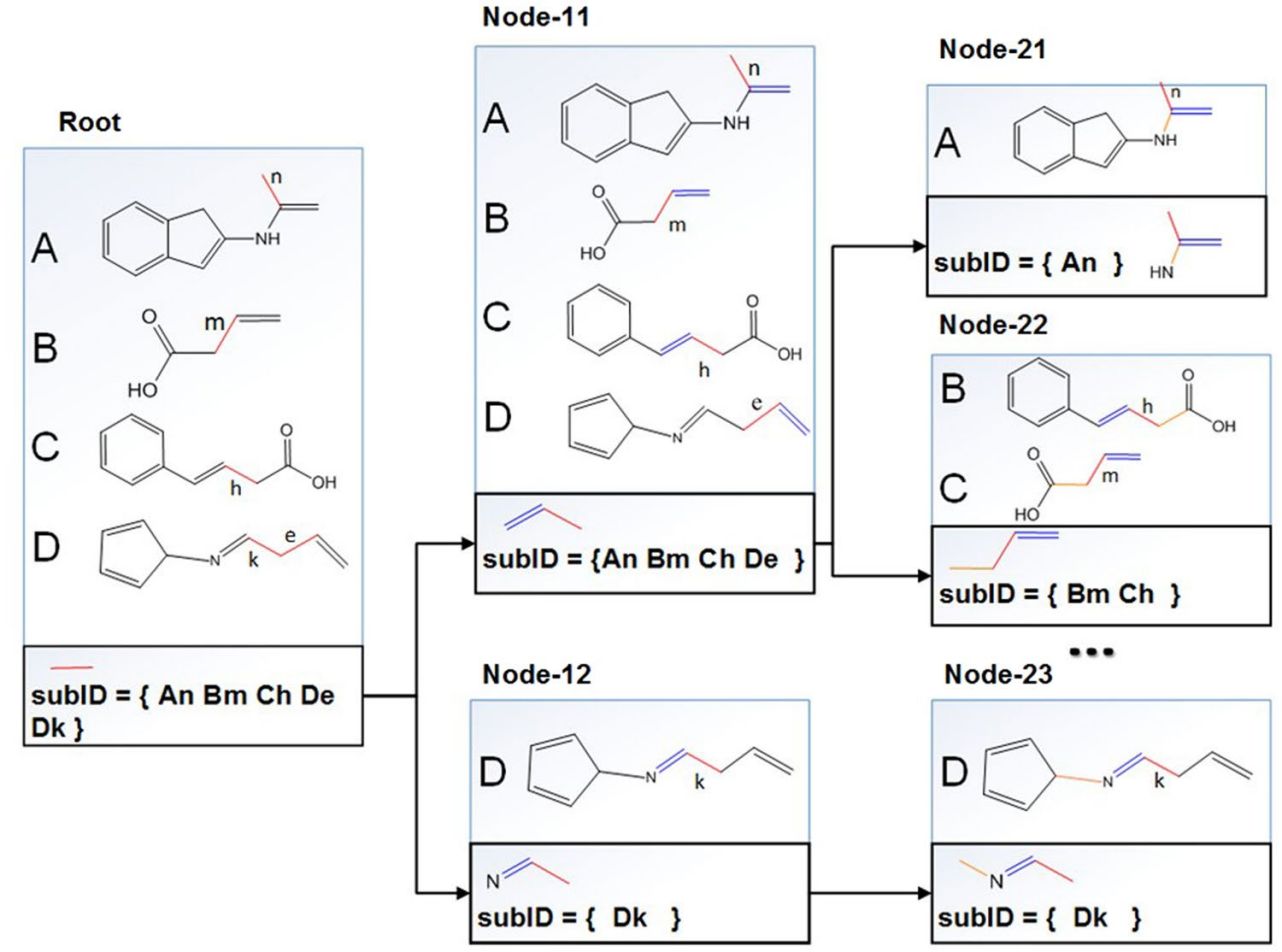
An example of a substructure generation tree. The tree started with a four compounds library with a C-C root fragment with a subID vector containing {An Bm Ch De Dk}. The popularity of this root fragment is 4. The root node produced two successor nodes (Node-11 and Node-12) by generating two new substructures. The process is repeated till all successor nodes are undividable. The substructures in the thick boxes are all possible fragment substructures created from a C-C root fragment. Other types of root fragments will be used to generate more substructure generation trees.

The tree is generated by a restricted depth-first search process, which only grows the node with the maximal substructure on the tree, other branches in the tree will be pruned. The tree starts with a single-edge fragments (for example, an edge with two carbon atoms connected in a single bond) called root fragments. Each substructure is expanded from a root fragment and is assigned with a subID (substructure identifier) vector. An element in the subID vector encodes the information of its parent molecule and root fragment. The tree grows by expanding root fragments through including adjacent edges (bonds). As shown in Fig. 16, the tree started with a root fragment of two carbon atoms with a single bond (subID = {An Bm Ch De Dk}). A subID consists of molecular IDs (denoted with capital letters) and bond IDs (denoted with lowercase letters). If a subID is generated from more than one molecule, the corresponding fragment is expanded and new nodes are added to the tree. In Fig. 1 at the root node of the tree, its subID consists of four members (popularity = 4). By expanding the fragment in the root node, two more nodes (Node-11 and Node-12) were added into the tree, and new subIDs were generated. The process was repeated till all successor nodes were undividable (subID consists only one member).

### Pruning a substructure generation tree

The tree can grow rapidly, and cause a serious “combinatorial explosion”, because a MG can have ${2}^{n}-1$ possible substructures, where *n* is the number of edges (chemical bonds, the chemical bonds with hydrogens are omitted). These substructures contain huge amount of redundant information that can be pruned to significantly reduce computing complexity and simplify substructure trees^37^. As shown in Fig. 16, The tree was generated from node **1**, and searched from the left branch (Node **1.1**). Since **1.1** was a leaf node, the algorithm kept searching on right branch (node **1.2**) until reached node **1.2.1.1.1.1.1**, which was termed as potential reporting node (PRN). A PRN node is defined as follows:

Let ***P*** be the subID component set for a parent node, ***C***_***1***_ ***, C***_***2***_, ***…C***_***n***_ be the subID component sets for the children nodes of the parent node (**n** is the number of the children nodes for PRN node).

Then, the parent node will be recognized as PRN node if (2) is satisfied:

2 $$\{(P\equiv {{\boldsymbol{C}}}_{{\boldsymbol{1}}})\vee (P\equiv {{\boldsymbol{C}}}_{{\boldsymbol{2}}})\vee \ldots \vee (P\equiv {{\boldsymbol{C}}}_{{\boldsymbol{n}}})\}={\rm{\Phi }}$$

For example, **1.2.1.1.1.1.1** (green box) was considered as a PRN because it had four children nodes and no child had the same as the subID of current node’s subID.

**Figure 17:**
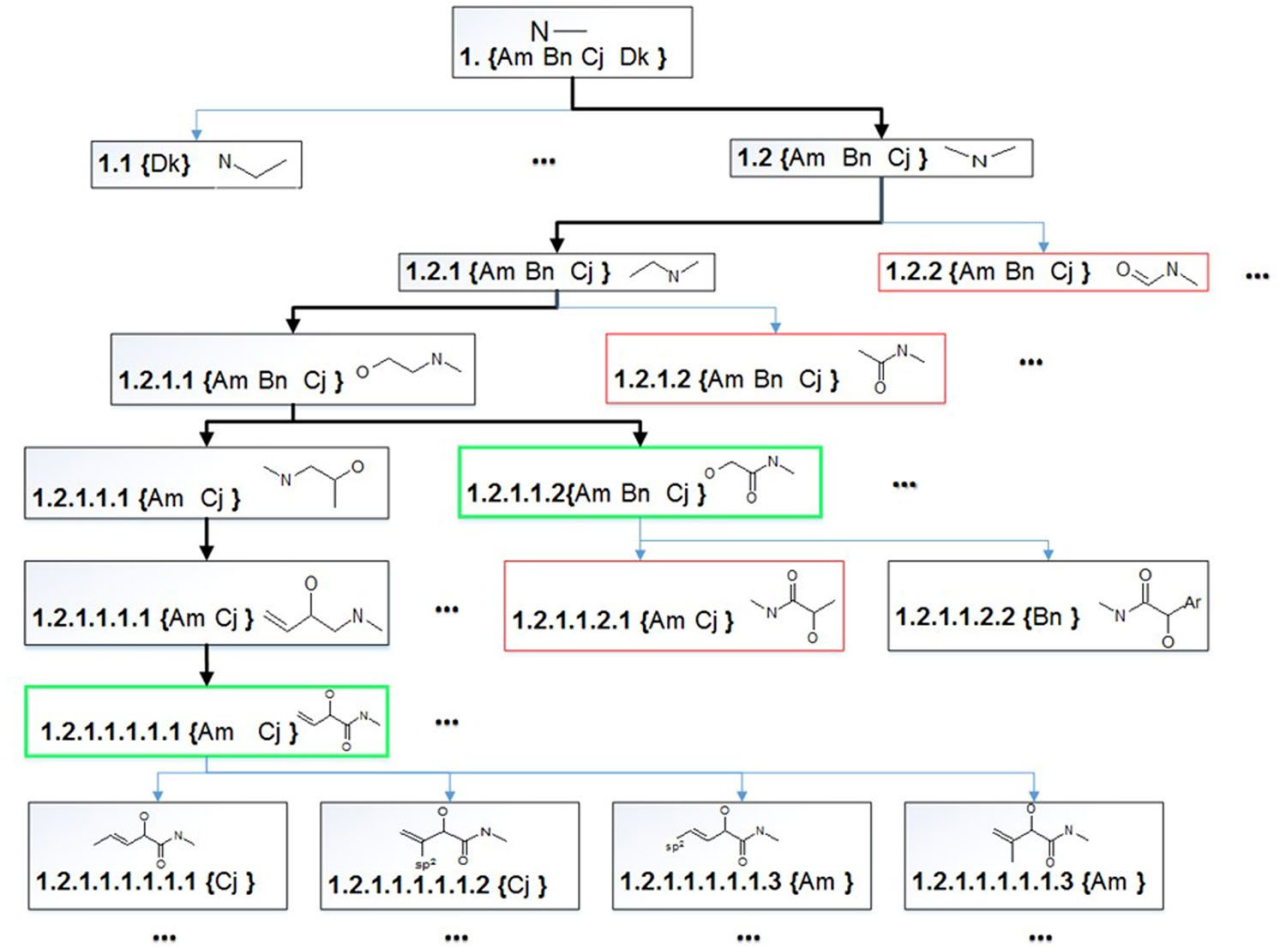
Pruning a substructure generation tree. The tree was growing while a depth-first search was proceeding. The nodes in black boxes were calculated on the fly. The nodes in green boxes were kept in an substructure library. The nodes in red boxes were pruned.

Each PRN had a popularity, which was the molecular counts encoded in subID. A substructure in PRN would be reported as a substructure if the PRN popularity was great than a designated threshold (*t* > 1), and the corresponding subID was recorded as well. Thus, a substructure library (containing subIDs and substructures) was generated and expanded when the tree was growing. When a new node was searched on the tree, its subID would be retrieved against the library. If the subID was found in the library, it would be pruned (red boxes in Fig. 17). Consequently, the successors of the pruned node would not be searched. The redundant information was avoided, and the computing complexity was significantly reduced.

**Figure 18:**
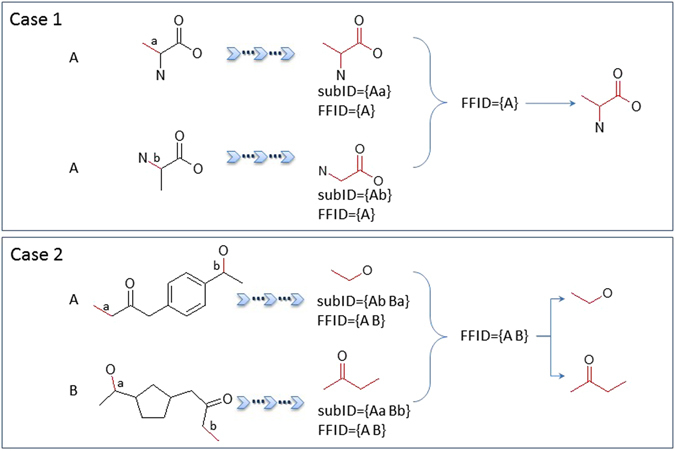
FFID and frequent substructures. Case 1: One FFID could have more than one fragments, one of the fragments was the substructure of another. The larger fragment was kept in this case. Case 2: One FFID could have more than one fragments, they were not topologically included to each other. Both fragments would be assigned to the same FFID.

Usually, a substructure, for example, the fragment in **1.2.1.1.1.1.1** node (Fig. 18), was the maximal substructure fragment (MSF) in a depth-first search path. After the MFS library was generated, the subIDs were converted into frequent fragment IDs (FFIDs), which came from subIDs by removing bondIDs. FFIDs encoded the information regarding their parents and popularities. Some FFIDs were assigned to unique fragments. Other FFIDs could have multiple fragments.

As shown in Fig. 18, if one FFID was assigned with two fragments, and if one is the substructure of another (checked by the substructure match algorithm^9^), then the smaller fragment was removed (Fig. 18 Case 1). The detailed implementation of this tree pruning strategy can be found in the supplementary material.

### Substructures as descriptors for a compound

QSAR study requires a descriptor vector for a compound. Each component in the descriptor vector is the count of a designated substructure that appeared in the compound. The designated substructures for the vector can be empirical (such as MDL 166 search keys or 960 extended search keys^32^, Daylight fingerprints^38^, or atom center fragments^13, 39^). In this work, we select the designated substructures for the vector based upon statistics. First, a SSD was derived from the ZINC database^31^, which contains more than 9.1 million chemical structures, to ensure the library covers known chemical diversity space. Let **SSD** have *n* substructures, a compound can be represented by a binary vector **BV** with *n* components, each component BV[i] (i ∈ *1..n*) has a value 0 or 1 for SSD[i] being absent or present in the compound structure, for further QSAR or classification studies.

### Data sets for classification and regression models using SSD

To examine the performance of the QSAR models using SSD, three data sets, LXRβ (Liver X receptor β), PPARα (peroxisome proliferator-activated receptor α), and VR (vasopressin receptor) libraries with chemical structures and bioactivities (IC_50_ values), were extracted from the BindingDB^40^. Duplicated structures in the libraries were filtered. Salt moieties in the connection tables were removed.

The activity data were pre-processed differently. For classification, the IC_50_ values were converted to zeros (in-actives) if they were greater than 10,000 nM, otherwise ones (actives). For regression, the records with the IC_50_ values, which were greater than 10,000 nM, were removed. Then, the IC_50_ values were converted to pIC_50_ values. This resulted in 717 and 634 LXRβ records, 784 and 621 PPARα records, and 619 and 491 VR records for classifications and regressions respectively.

The structural descriptors were selected from the SSD based upon their appearances in the corresponding data set. The frequency of a structural descriptor in a data set less than 5% was not selected.

### Regularized logistic regression (RLR) method for compound classification

A compound library with known bioactivity results is represented in a matrix ***L***[*1..n*, *1*..*m*], where *n* is the number of the substructures selected from the SSD, and *m* is the number of compound structures in the library. The bioactivity data of the library is represented with **A**[*1..m*]. RLR^41^ will figure out parameter vector ***W***[*1..m*] in (3).

3 $${\boldsymbol{f}}({{\boldsymbol{L}}}_{{\boldsymbol{j}}}\cdot {\boldsymbol{W}})={A}_{{\boldsymbol{j}}}$$

***L***_*j*_ is the descriptors for the *jth* compound, *A*_*j*_ is the predicted activity (0 or 1) for the *jth* compound. Let **F** stand for **SSD**, **X** stand for the structures in the compound library ***L***, ***S*** *[1..m]* stand for the scores of compounds being active, then, *L[i, j]*, an element of **L** is defined in (4),

4 $$L[i,\,j]=\{\begin{array}{l}1\,SMFSD[i]\in X[j]\,\\ 0\,\neg (SMFSD[i]\in X[j])\end{array}$$

where *SMFSD[i]* is the *ith* MFS in **SMFSD**, and *X[j]* is the *jth* compound in **L**.

According to RLR approach^41^, the bioactive probability of the *ith* compound can be calculated in (5),

5 $$p({A}_{j}=1{{\boldsymbol{L}}}_{j},{\boldsymbol{W}})=\frac{{e}^{{\boldsymbol{W}}{\boldsymbol{\bullet }}{{\boldsymbol{L}}}_{j}}}{1+{e}^{{\boldsymbol{W}}{\boldsymbol{\bullet }}{{\boldsymbol{L}}}_{j}}}$$

and the non-bioactive probability of the *ith* compound can be calculated in (6),

6 $$p({A}_{j}=0|{{\boldsymbol{L}}}_{j},{\boldsymbol{W}})=\frac{1}{1+{e}^{{\boldsymbol{W}}\cdot {{\boldsymbol{L}}}_{j}}}$$

where S[j] is the activity prediction for the *jth* compound in **L**.

Machine learning process is to figure out ***W*** by optimizing (7) and (8) through logic regressions. For the *jth* compound,

7 $$\mathrm{log}(p({A}_{j},\,{\boldsymbol{W}}|{{\boldsymbol{L}}}_{j})=\,\mathrm{log}(p({A}_{j}|{{\boldsymbol{L}}}_{j},{\boldsymbol{W}})-\frac{1}{2\sigma }\Vert {\boldsymbol{W}}\Vert +C$$

where σ is standard deviation, *C* is a constant.

For all compounds,

8 $${\rm{L}}({\bf{W}})=\sum _{j=1}^{m}\,\mathrm{log}(p({A}_{j}|{L}_{j},{\boldsymbol{W}})-\frac{m}{2}\Vert {\boldsymbol{W}}\Vert +C$$

We obtain optimized values for W through Newton iteration method^29^, because the second gradient of L(**W**) is always greater than zero.

### Evaluating SSD-based classification results

ROC and following parameters were calculated to evaluate the MFS-based classification approach^42^.

SSD-based models were validated with the random sub-sampling cross validation method^43^. Initially, each experimental data set was randomly divided into 3 subsets; randomly selected 2 subsets to train the models, and the remaining subset was used for validating the models. The validating parameters were calculated and averaged over each batch of validations.

### Predicting activities with support vector machines (SVM) using the substructure discriptors

In a SVM regression model^44^, the IC_50_ was converted to pIC_50_ (−log(IC_50_)), which is proportional to the bioactivity. pIC_50_ is the function of the descriptors, *f(L*_*x*_), and was calculated as the following:

9 $$f({{\boldsymbol{L}}}_{i})=\,\sum _{j=1}^{n}{{\rm{\alpha }}}_{j}{\rm{k}}({{\boldsymbol{L}}}_{j},{{\boldsymbol{L}}}_{i})+b$$

where ***L***_*i*_ is the descriptors (FS) of the *ith* compound; *n* is the number of the subsets (support vectors) from a training set; *j* is a compound in the subset; α_*j*_ is the regression parameter for the *jth* compound; *b* is a regression constant to be determined by SVM regression process; k is a RBF kernel function defined in (10),

10 $${\rm{k}}({{\boldsymbol{L}}}_{i},{{\boldsymbol{L}}}_{j})=\exp (\frac{-{\Vert {{\boldsymbol{L}}}_{i}-{{\boldsymbol{L}}}_{j}\Vert }^{2}}{2{\sigma }^{2}})$$

where σ is the standard deviation.

### SVM model evaluation method

The SVM models were cross-validated through the average mean square error (MSE) and Pearson correlation of predicted and experimental pIC_50_ values with a k-fold cross-validation approach.

### Method for selecting privileged substructure from SVM models

A privileged substructure is the one that is responsible for desired activities. Privileged substructures were derived from SVM models by ranking them with their *p*-values(*p*). If a MF substructure were used in a SVM model, its *p*-value (the function of the observed sample results that is used for testing a statistical hypothesis) was calculated with one-tailed test Fisher’s exact test^45^. Let *A* and *B* be the numbers of matched and unmatched substructures for the *ith* substructure in an active molecule from a training set; let *C* and *D* be the numbers of matched and unmatched substructures for *ith* substructures in a molecule from a background set (in our case, it is ZINC compound library). The *p*-value (*p*_*i*_) is calculated as the following:

11 $$\,{p}_{i}=\sum _{a,b,c,d}\frac{(\begin{array}{c}a+b\\ a\end{array})\,(\begin{array}{c}c+d\\ c\end{array})\,}{(\begin{array}{c}a+c+d+b\\ a+c\end{array})}\,$$

where *a* and *b* are numbers of matched and unmatched substructures for the *ith* substructure in an active molecule from a training set; and *c* and *d* are numbers of matched and unmatched substructures for *ith* MF substructures in a molecule from a background set. In an active data set. And, $\frac{a}{c} > \frac{A}{C}$, *a* + *b* = *A* + *B*, *c* + *d* = *C* + *D*. The *p*-values were adjusted with false-discovery rate (FDR) approach^46^.

The substructures were sorted in the ascending orders of *p*-values. The significant substructures are with *p*-values < 0.05. Privileged substructures were elected by using high-scored substructures as substructure queries searching against the targeted compound library. The hits with a high number of active compounds were identified as the privileged substructures of the focused library.

### Deriving the rules of combining fragments

Let A = {a[0], a[1], … a[x], …a[M − 1]} as a privileged substructure list derived from a compound library; B = {b[0], b[1], … b[y], … b[N − 1]} as the compound list. The rules for combining fragments for FBDD study can be discovered in the following pseudo-code:

## Electronic supplementary material

Supplementary Information 
