# Supplementary material for: A de novo substructure generation algorithm for identifying the privileged chemical fragments of liver X receptorβ agonists
Source: Sci Rep. 2017 Sep 11;7:11121. doi: 10.1038/s41598-017-08848-4 (PMC5593923; doi:10.1038/s41598-017-08848-4)
Supplement: Supplementary file 1 — Supplementary Information [file 41598_2017_8848_MOESM1_ESM.pdf]

A *de novo* substructure generation algorithm for identifying the privileged chemical fragments of liver X receptorβ agonists

He Peng‡, Zhihong Liu‡, Xin Yan, Jian Ren* and Jun Xu*

Research Center for Drug Discovery, School of Pharmaceutical Sciences and School of Life Sciences, Sun Yat-Sen University, 132 East Circle at University City, Guangzhou 510006, China

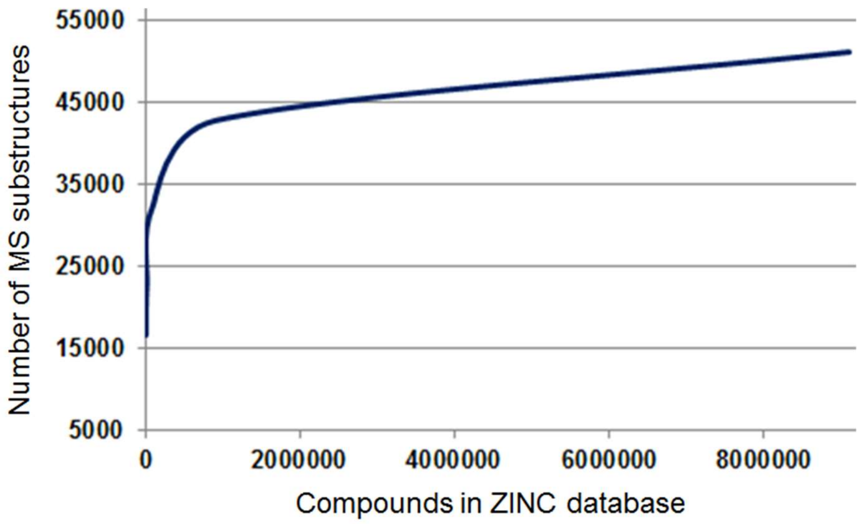

Figure S1. The number of substructures was approaching a limit when the DSGA was applied to a large compound library (ZINC database).

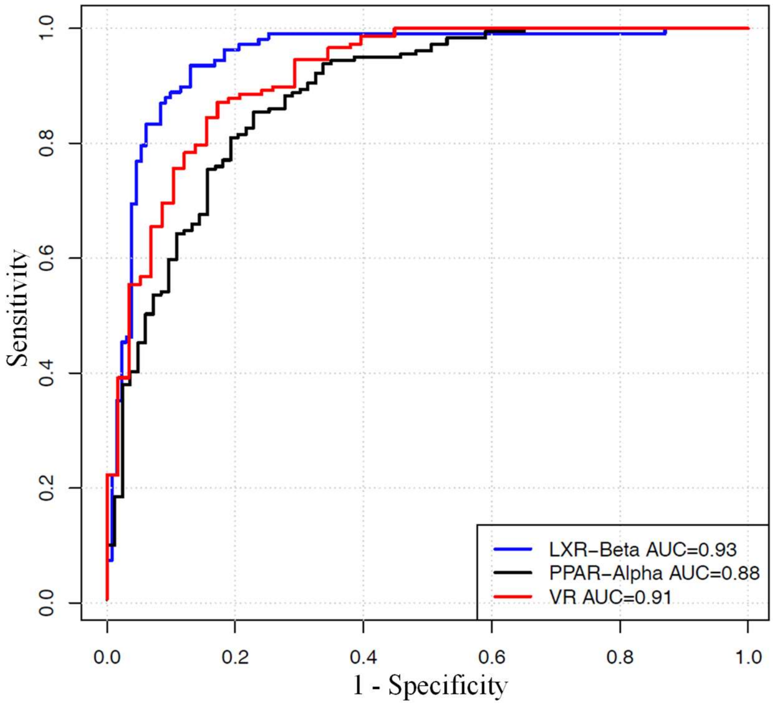

Figure S2. The ROC curves of the three classification models against three drug targets.

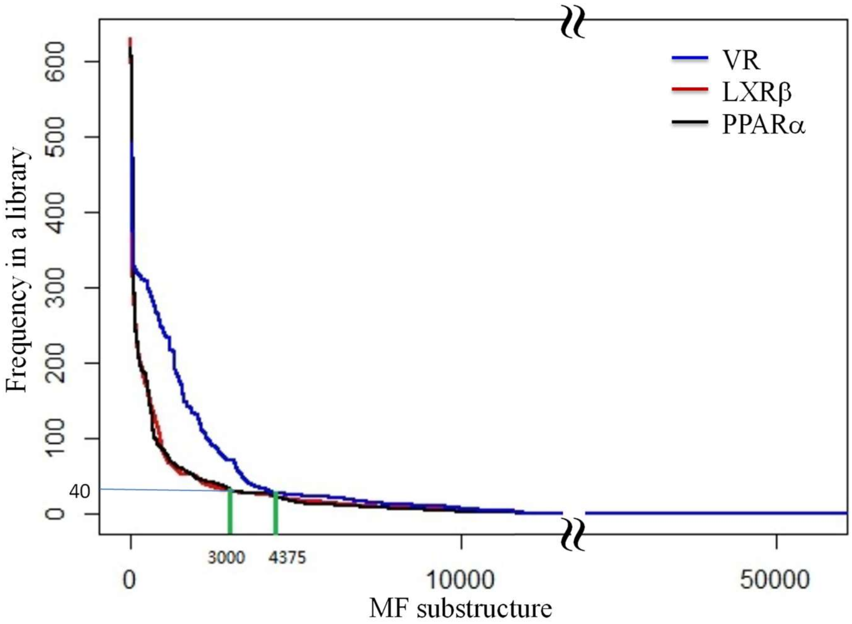

Figure S3. The SSD substructure distributions for the VR, LXR□, and PPAR□ libraries. The turning points are at the frequency of 40.

SM Table 1. Ligands made through Rule 1.

LARβ ligand	Linker	EC_{50}(μM)
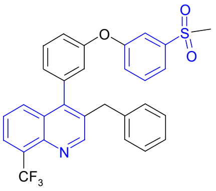	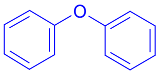	β: 0.011<br>α: na
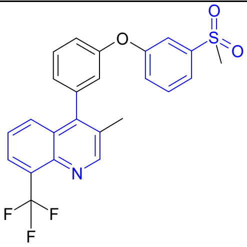	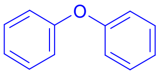	β: 0.031<br>α: 0.183
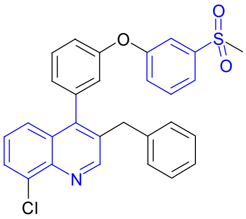	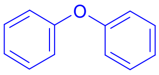	β: 0.030<br>α: na
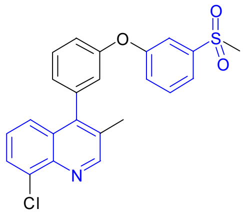	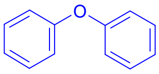	β: 0.044<br>α: na
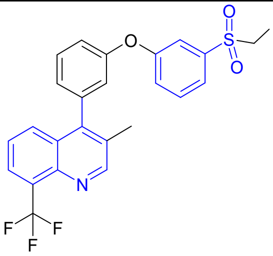	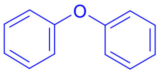	β: 0.055<br>α: na
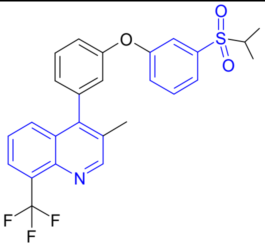	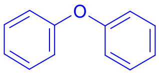	β: 0.056<br>α: na

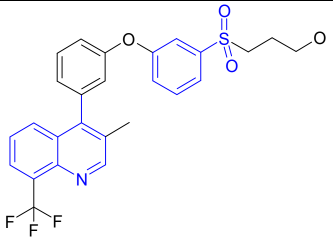	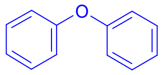	β: 0.070<br>α: na
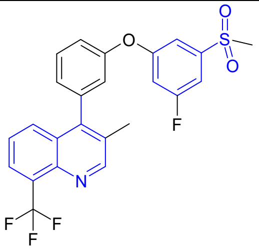	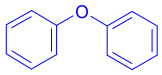	β: 0.090<br>α: na
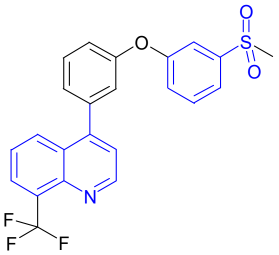	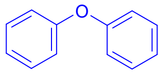	β: 0.214<br>α: na
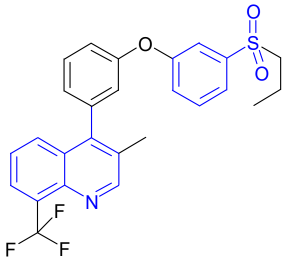	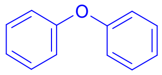	β: 0.251<br>α: na
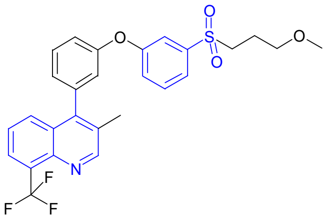	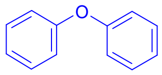	β: 0.274<br>α: na
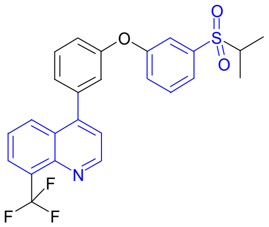	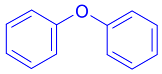	β: 0.299<br>α: na

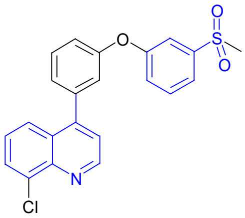	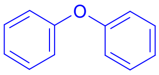	β: 0.382<br>α: na
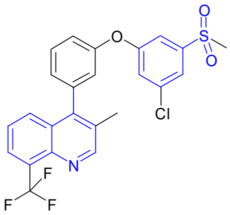	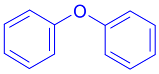	β: 0.445<br>α: na
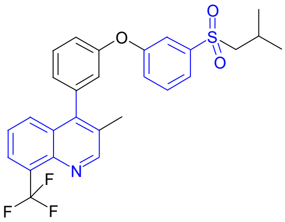	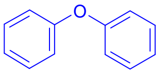	β: 0.592<br>α: na
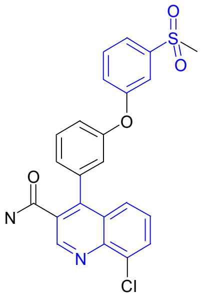	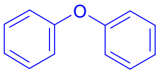	β: 0.630<br>α: na
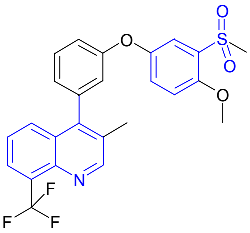	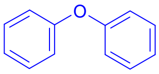	β: 0.688<br>α: na
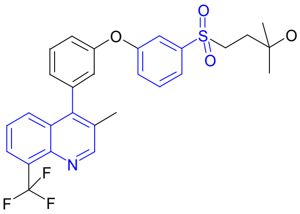	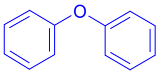	β: 0.962<br>α: na

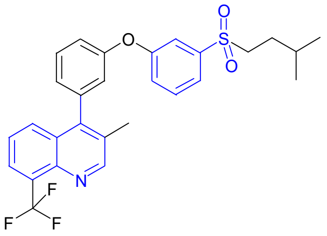	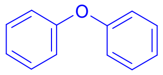	β: 1.030<br>α: na
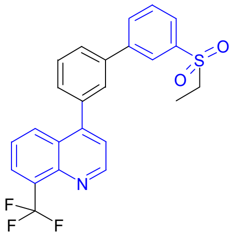	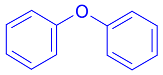	β: 1.080<br>α: na
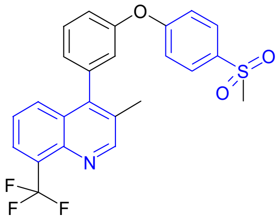	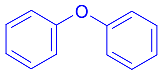	β: 1.155<br>α: na
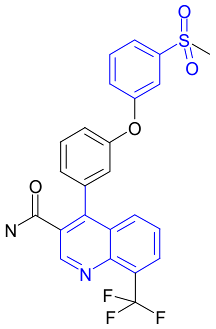	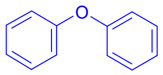	β: 1.600<br>α: na
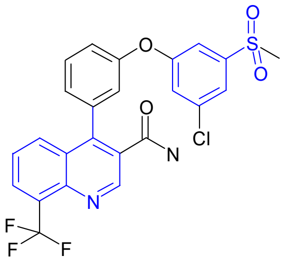	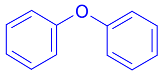	β: 1.700<br>α: 5.100

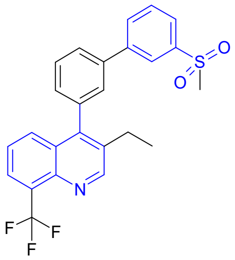	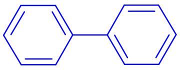	β: 0.076<br>α: na
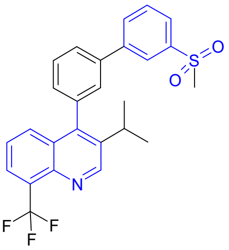	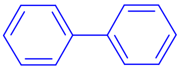	β: 0.091<br>α: na
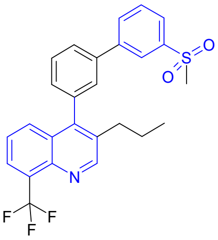	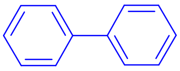	β: 0.106<br>α: na
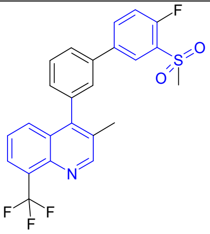	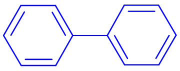	β: 0.161<br>α: na
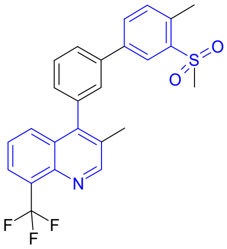	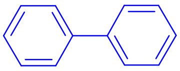	β: 0.175<br>α: na
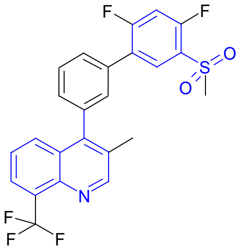	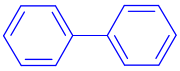	β: 0.195<br>α: na

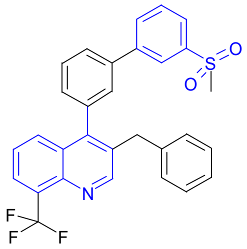	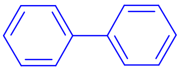	β: 0.100<br>α: na
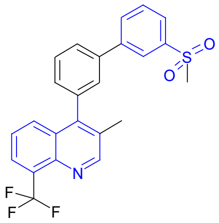	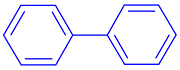	β: 0.284<br>α: na
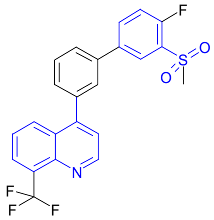	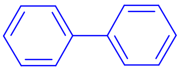	β: 0.512<br>α: na
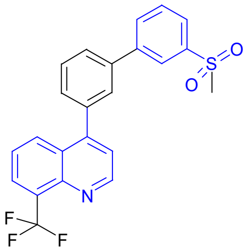	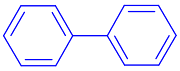	β: 0.525<br>α: na
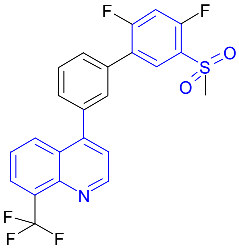	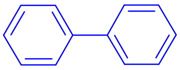	β: 0.565<br>α: na
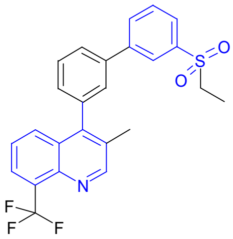	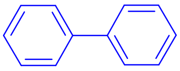	β: 0.580<br>α: na

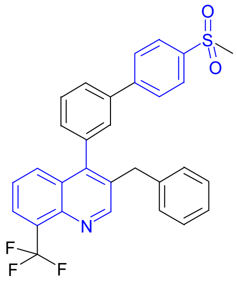	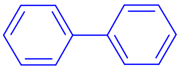	β: 3.300<br>α: na

SM Table 2. Ligands made through Rule 2 (Fragment A + Fragment C).

LARβ ligand	Fragment C	EC_{50}(μM)
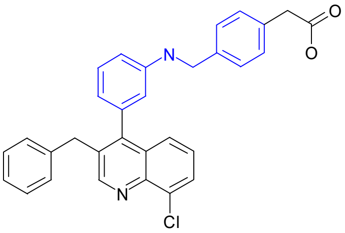	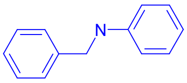	β: 0.023<br>α: na
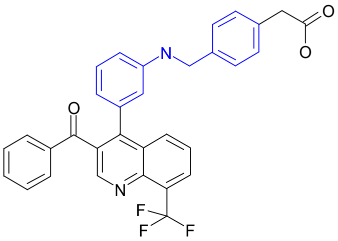	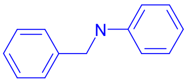	β: 0.029<br>α: na
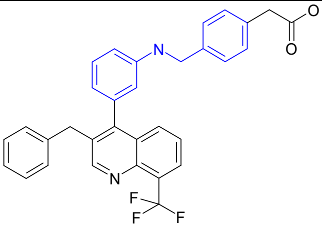	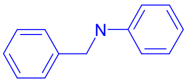	β: 0.033<br>α: na
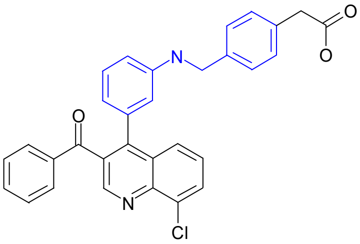	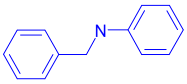	β: 0.039<br>α: na

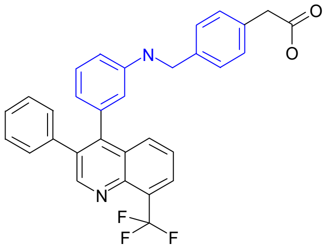	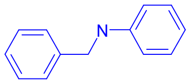	β: 0.044<br>α: na
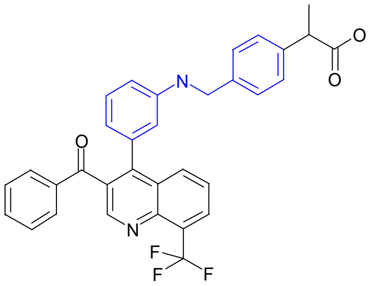	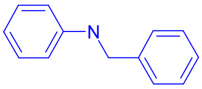	β: 0.058<br>α: na
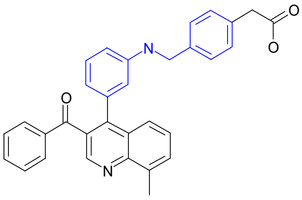	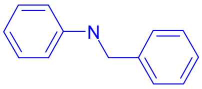	β: 0.061<br>α: na
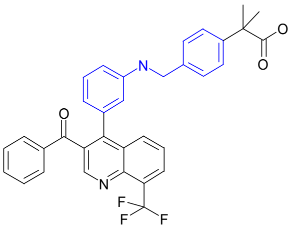	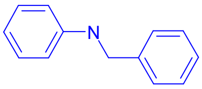	β: 0.078<br>α: na
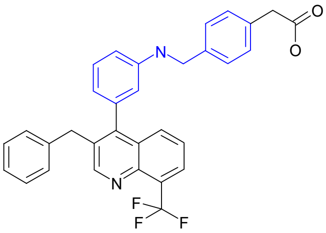	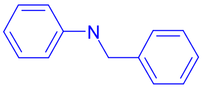	β: 0.090<br>α: 0.160
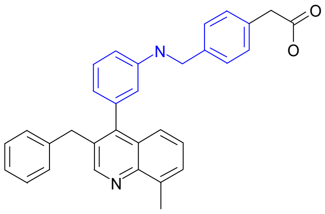	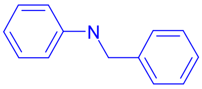	β: 0.090<br>α: na

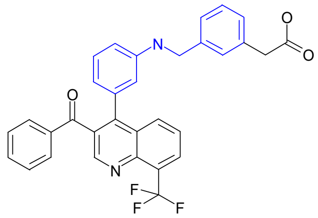	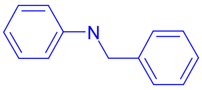	β: 0.1410<br>α: na
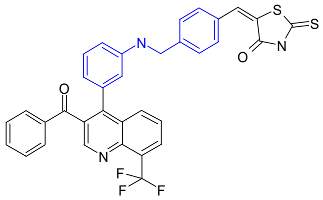	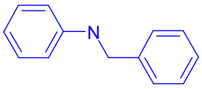	β: 0.2860<br>α: na
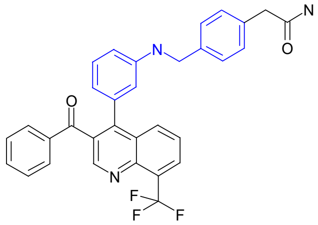	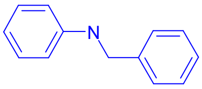	β: 0.3160<br>α: na
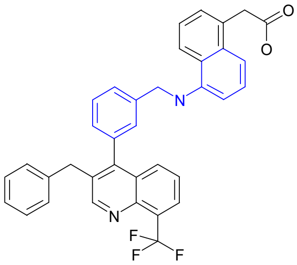	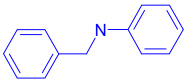	β: 0.3550<br>α: na
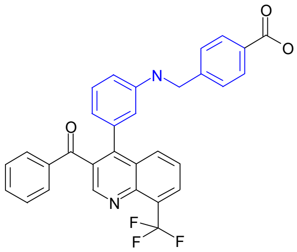	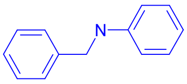	β: 0.3650<br>α: na
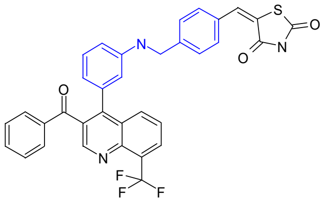	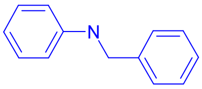	β: 0.3690<br>α: na

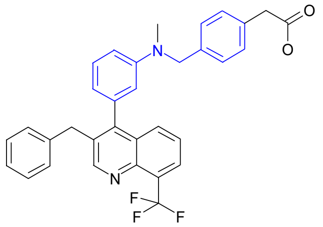	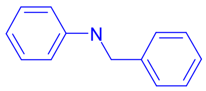	β: 0.3740<br>α: na
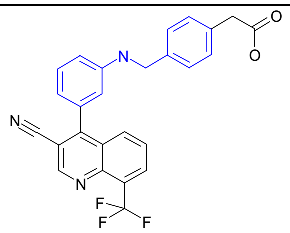	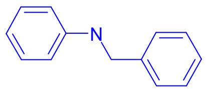	β: 0.4500<br>α: na
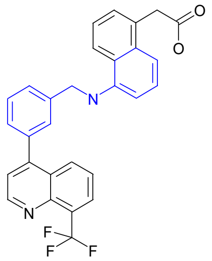	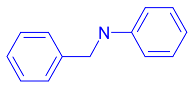	β: 0.6220<br>α: na
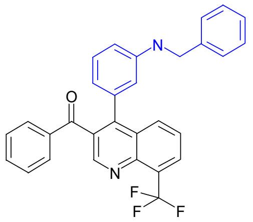	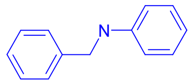	β: 0.7700<br>α: na
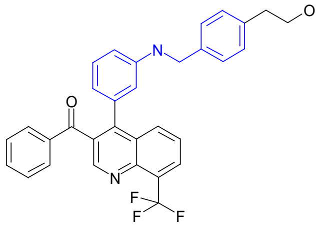	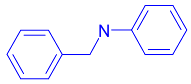	β: 0.7950<br>α: na
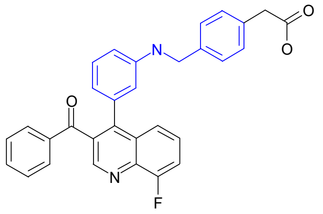	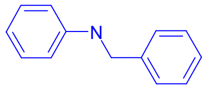	β: 0.7000<br>α: na

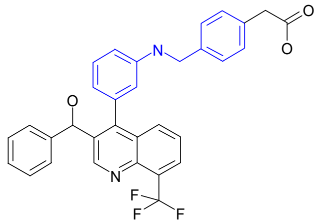	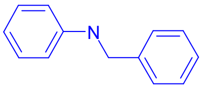	β: 0.8120<br>α: na
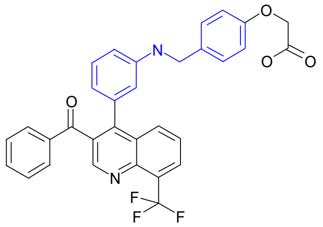	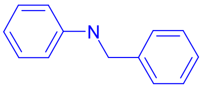	β: 1.2710<br>α: na
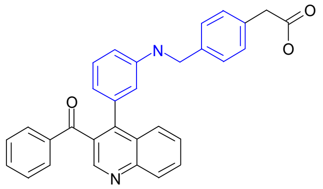	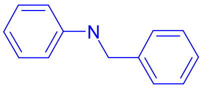	β: 1.6500<br>α: na
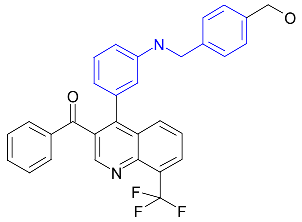	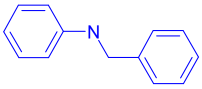	β: 2.30400<br>α: na
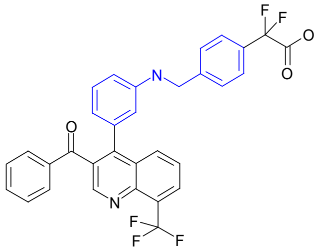	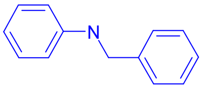	β: 2.39500<br>α: na
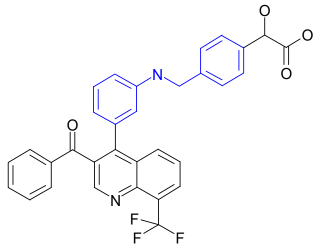	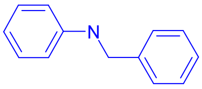	β: 4.0030<br>α: na

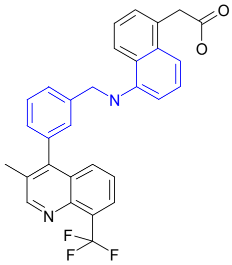	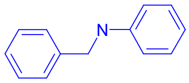	β: 4.1670<br>α: na
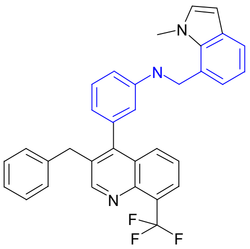	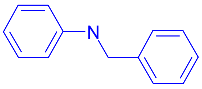	β: 4.1700<br>α: na
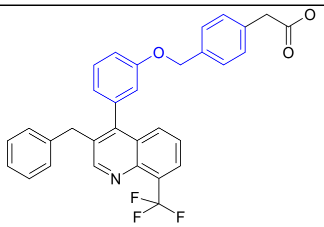	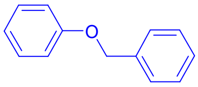	β: 0.071<br>α: na
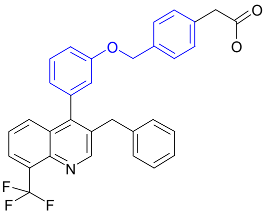	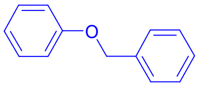	β: 0.093<br>α: na
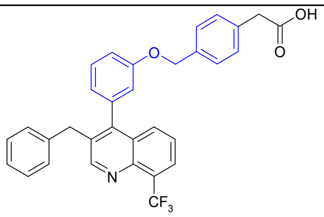	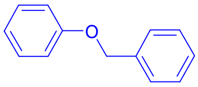	β: 0.090<br>α: 0.240
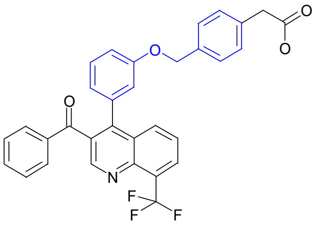	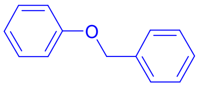	β: 0.1430<br>α: na

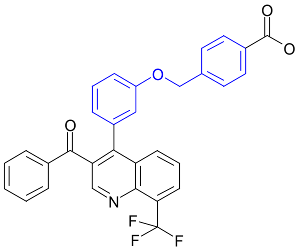	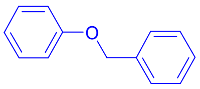	β: 0.1860<br>α: na
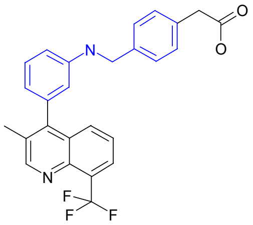	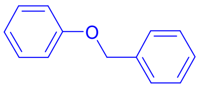	β: 0.1000<br>α: na
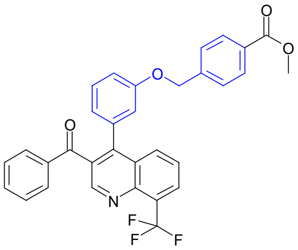	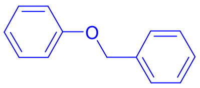	β: 0.2960<br>α: na
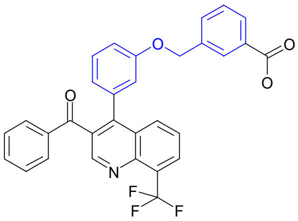	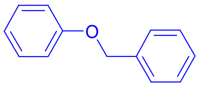	β: 1.500<br>α: na
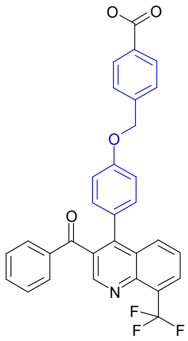	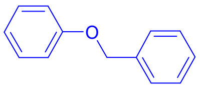	β: 1.500<br>α: na

SM Table 3. The typical LXR agonists for Rule 4.

ID	structure	EC50beta	EC50alpha

380	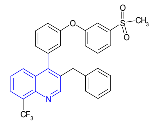	0.011uM	
41	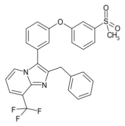	0.020uM	0.144uM
31	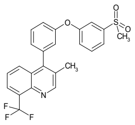	0.031uM	0.183uM
46	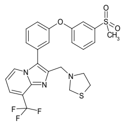	0.032uM	0.170uM
382	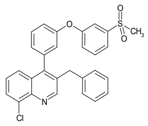	0.03uM	
383	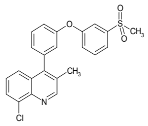	0.044uM	
385	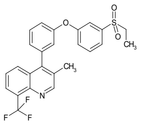	0.055uM	
387	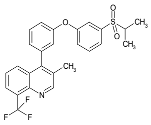	0.056uM	
391	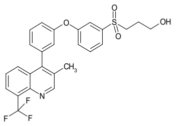	0.07uM	
395	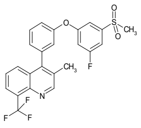	0.09uM	
	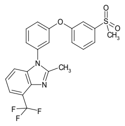		

32		0.162uM	0.64uM
45	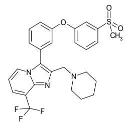	0.204uM	0.92uM
381	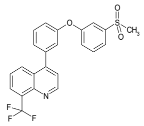	0.214uM	
386	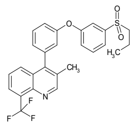	0.251uM	
393	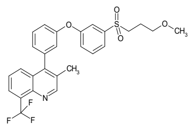	0.274uM	
388	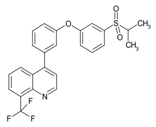	0.299uM	
384	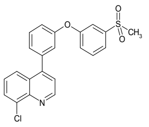	0.382uM	
37	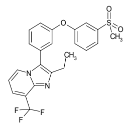	0.42uM	1.22uM
396	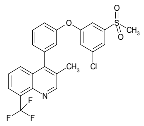	0.445uM	
54	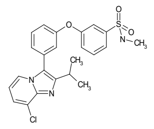	0.53uM	1.61uM

48	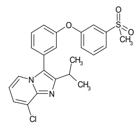	0.54uM	1.66uM
40	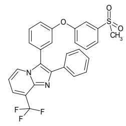	0.57uM	1.11uM
389	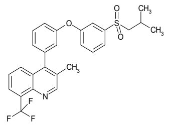	0.592uM	
240	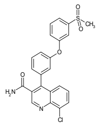	0.63uM	3.0uM
394	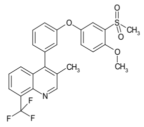	0.688uM	
34	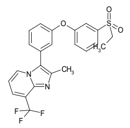	0.68uM	2.28uM
35	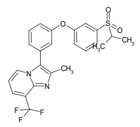	0.70uM	4.1uM
39	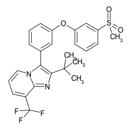	0.71uM	2.18uM
33	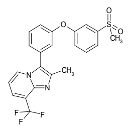	0.84uM	2.54uM
47	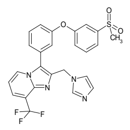	0.85uM	1.7uM

52	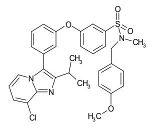	0.89uM	0.93uM
38	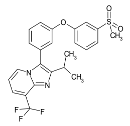	0.95uM	1.86uM
392	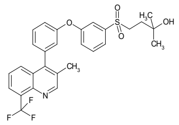	0.962uM	
390	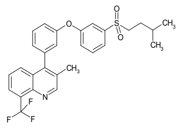	1.03uM	
36	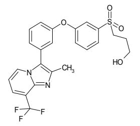	1.42uM	6.0uM
378	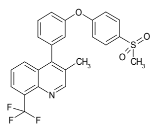	1.550uM	
56	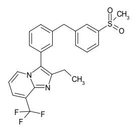	1.57uM	3.2uM
49	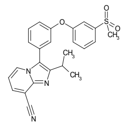	1.68uM	3.9uM
239	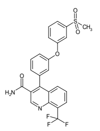	1.6uM	4.5uM
244	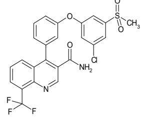	1.70uM	5.10uM

SM Table 4. The typical LXR agonists for Rule 5.

ID	structure	EC50beta	EC50alpha
104	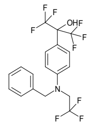	0.2uM	0.9uM
105	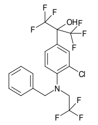	0.02uM	0.1uM
106	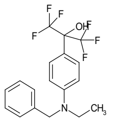	0.4uM	1.1uM
107	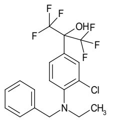	0.08uM	0.2uM
121	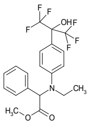	2.2uM	2.3uM
122	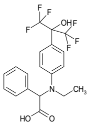	5.4uM	9.4uM
123	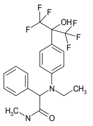	11uM	10.9uM
124	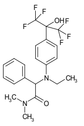	2.3uM	9.3uM
125	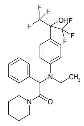	2.2uM	2.2uM
126	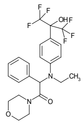	2.3uM	8.5uM
127	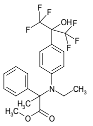	0.03uM	0.1uM
128	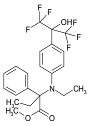	0.07uM	0.3uM

129	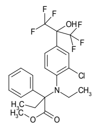	0.07uM	0.5uM
130	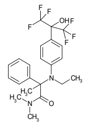	0.07uM	0.4uM
131	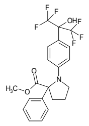	0.04uM	0.2uM
132	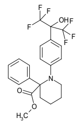	0.04uM	0.09uM
134	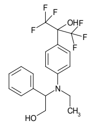	0.7uM	1.9uM
135	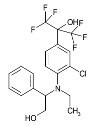	0.2uM	0.4uM
136	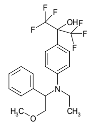	0.1uM	0.9uM
137	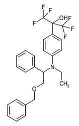	0.6uM	1.9uM
138	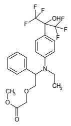	0.9uM	2.2uM
140	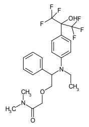	0.26uM	0.8uM
141	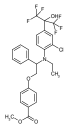	0.2uM	0.7uM
142	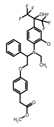	0.3uM	1.1uM

143	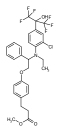	0.6uM	1.4uM
144	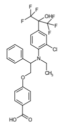	1.1uM	3.7uM
145	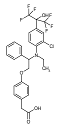	2.4uM	5.5uM
146	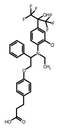	1.2uM	4.5uM
246	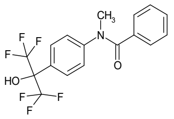	0.18uM	0.63uM
247	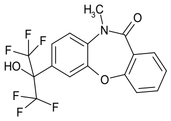	2.7uM	4.2uM
248	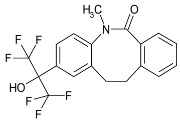	0.26uM	0.91uM
249	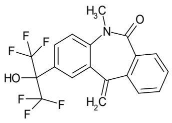	0.77uM	1.7uM
250	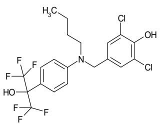	3.1uM	7.1uM
271	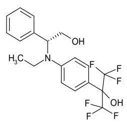	0.4uM	1.5uM
272	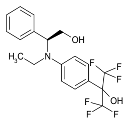	0.1uM	0.5uM
453	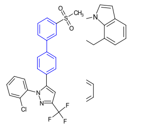	0.049uM	

454	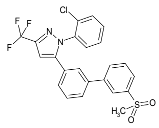	0.23uM
456	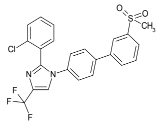	0.061uM
457	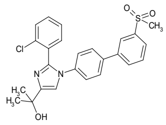	0.6uM
458	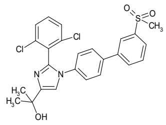	0.67uM
